# Sexual Dimorphism through the Lens of Genome Manipulation, Forward Genetics, and Spatiotemporal Sequencing

**DOI:** 10.1093/gbe/evaa243

**Published:** 2020-11-18

**Authors:** Katja R Kasimatis, Santiago Sánchez-Ramírez, Zachary C Stevenson

**Affiliations:** 1 Department of Ecology and Evolutionary Biology, University of Toronto, Ontario, USA; 2 Institute of Ecology and Evolution, University of Oregon, Eugene, Oregon, USA

**Keywords:** sexual antagonism, experimental evolution, CRISPR, single-cell sequencing, long-read sequencing

## Abstract

Sexual reproduction often leads to selection that favors the evolution of sex-limited traits or sex-specific variation for shared traits. These sexual dimorphisms manifest due to sex-specific genetic architectures and sex-biased gene expression across development, yet the molecular mechanisms underlying these patterns are largely unknown. The first step is to understand how sexual dimorphisms arise across the genotype–phenotype–fitness map. The emergence of “4D genome technologies” allows for efficient, high-throughput, and cost-effective manipulation and observations of this process. Studies of sexual dimorphism will benefit from combining these technological advances (e.g., precision genome editing, inducible transgenic systems, and single-cell RNA sequencing) with clever experiments inspired by classic designs (e.g., bulked segregant analysis, experimental evolution, and pedigree tracing). This perspective poses a synthetic view of how manipulative approaches coupled with cutting-edge observational methods and evolutionary theory are poised to uncover the molecular genetic basis of sexual dimorphism with unprecedented resolution. We outline hypothesis-driven experimental paradigms for identifying genetic mechanisms of sexual dimorphism among tissues, across development, and over evolutionary time.

**Significance**

Sexual dimorphism is ubiquitous in sexually reproducing species and appears to be driven by widespread patterns of sex-biased gene expression. However, we do not understand how genetic material shared between the sexes creates these patterns or how sex-limited and sexually antagonistic selection shape the evolution of sexual dimorphism in genomes. We propose that this knowledge gap can be addressed by adapting tools from molecular genetics and biomedical research to an evolutionary genetics framework. We highlight experimental paradigms for identifying the genetic architecture of sexually dimorphic phenotypes and 4D genomic technologies for isolating the molecular mechanisms generating sexual dimorphism.

## Introduction

A central goal of evolutionary genetics is to understand the genetics of adaptation. This goal requires researchers to probe the genomic response to selection on phenotypes with a known fitness effect in nature (Barrett and Hoekstra 2011). We can approach this challenging task by studying distinct components of the problem: mapping the genetic basis of the phenotype, measuring selection on the phenotype, and scanning the genome for signatures of this selection. Sexual dimorphism (SD) of phenotypes adds an additional layer of complexity, because the sexes maximize fitness differently and are subject to different selective pressures (Trivers 1972; Parker 1979; Arnqvist and Rowe 2005). Yet, the sexes share the majority of their genetic material and, thus, SD is a function of shared, and of sex-specific and sex-biased genetic architecture, gene regulation, and gene expression. Therefore, to truly understand how sex-specific selection shapes the evolution of SD in genomes, it is essential to identify the molecular biology processes linking the genome with the phenome (box 1).

Identifying the genetic variants and sex-biased networks underlying SD has proved challenging. In the last decade, research has centered on patterns of sex-biased gene expression, which has led to the identification of strong, consistent sex-biases coupled with rapid molecular evolution and genomic organization of sex-biased genes (Reinke et al. 2000; Jin et al. 2001; Ranz et al. 2003; Cutter and Ward 2005; Yang 2006; Innocenti and Morrow 2010; Bohne et al. 2014; Harrison et al. 2015). Although informative, these global patterns mask the underlying molecular mechanisms and often do not directly provide spatial resolution within the organism. These limitations hinder our understanding of how SD is cued within tissues and across developmental time. Finally, the focus on transcriptional patterns alone excludes other sources of phenotypic variation such as translation.

Combining molecular genetics with classic evolutionary approaches and genome technology, provides an opportunity to uncover the molecular mechanisms linking a sexually dimorphic phenotype with its underlying genetic basis. Such integration is feasible, efficient, and cost-effective in the emerging era of “4D genome technologies” and can provide high-resolution analyses of biological features in distinct physiological and tissue systems, and across developmental and evolutionary time. Using this integrated evolutionary framework, we can begin to address long-standing questions in the field, such as: What is the genetic architecture of sexually dimorphic traits? What are the genetic constraints on sexual dimorphism? What is the relationship between sex chromosome evolution and sexual dimorphism? And, when, where, and how are sex-biased networks formed and how are they sustained across an organism’s lifecycle? This perspective aims to provide a synthetic view of how 4D genome technologies integrated into evolutionary frameworks can uncover the mechanistic basis and genomic manifestation of SD with unprecedented detail. We suggest that these new paradigms will overcome an emerging recognition of limitations to existing approaches for deciphering signals of SD and sexually antagonistic selection.

## Approaching Outstanding Questions

To map the genetic basis and molecular mechanisms of a sexually dimorphic phenotype, we can manipulate selection, correlate genomic patterns with SD and genetic sex, and verify the functional importance of genes through genomic manipulation. We briefly explore five complementary experimental paradigms and highlight how they can link genotype, phenotype, and fitness across the lifecycle of each sex to provide the maximum temporal resolution of the genetic basis and molecular mechanisms of SD.

### Evolve and Resequence

**Fig. 1:**
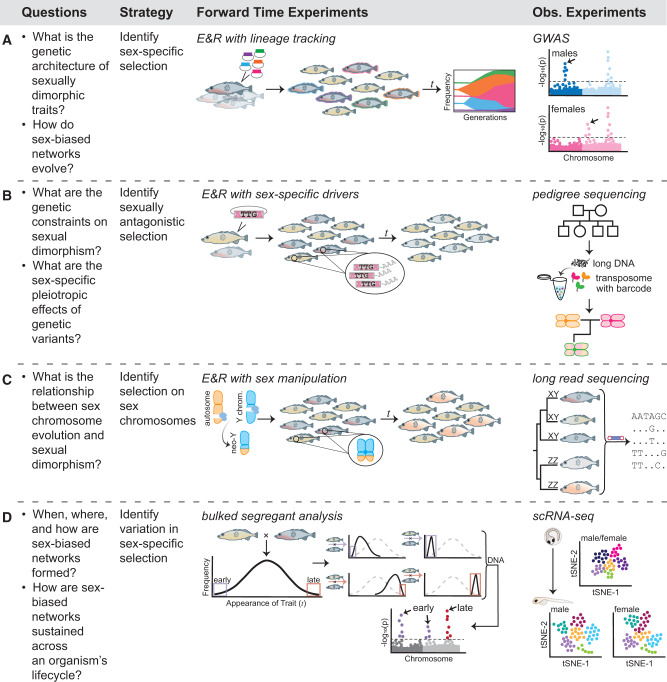
Evolutionary frameworks for identifying the genetic basis of SD. Strategies for identifying: (*A*) the genetic basis of SD through experimental evolution and between-sex comparative genomics, (*B*) sexual antagonism and SD through experimental evolution and pedigree tracing, (*C*) sex chromosome directed SD through experimental evolution and comparative genomics, and (*D*) developmental SD through bulked segregant analysis and single-cell sequencing.

The evolve and resequence (E&R) approach (Schlötterer et al. 2015) combines experimental evolution with whole-genome sequencing to trace allele frequencies over tens or hundreds of generations (fig. 1*A–C*). Examining allele frequency changes permits an estimation of the strength of selection on regions of the genome that contribute to the SD of interest. E&R is a powerful approach to manipulate selection in a sex-specific manner to examine sex-biased genetic architecture and determine: if there are genomic hotspots of SD, the relative contribution of coding versus regulatory sequence, and the number of contributing loci. Experimental evolution approaches have successfully isolated sex-specific selection (Rice 1996) and sexual selection (Chenoweth et al. 2008; Maklakov et al. 2009; Edward et al. 2010; Snook et al. 2013; Rostant et al. 2020), though few studies have examined the genomic response (see Hsu et al. 2020). New transgenic technology will expand the potential of E&R to identify sex-biased elements of genetic architecture by creating high-throughput mechanisms for altering the variance in or manipulating the developmental timing of a sexually dimorphic phenotype, or isolating selection to a given sex. For example, introducing inducible knockdown technology (supplementary table 1, Supplementary Material online) into the genome prior to E&R can provide a fine-scale experimental tool to alter gene expression in a sex-specific manner. By manipulating gene expression, the phenotypic mean can be shifted toward more or less SD, which will affect the response to selection. Inducible technology can aid in altering gene expression or the timing of gene expression, both of which will affect the sex-specific response to selection. Limiting selection to act within one sex during E&R will also be aided by tools that remove a phenotype in one sex, such as inducible sterility (Kasimatis et al. 2018), or generate progeny of a single sex (Douglas et al. 2020).

Alternatively, transgenic technology can expand our understanding of the genomic response during E&R through haplotype barcoding. This tool would provide a method to track the frequency of individual haplotypes (fig. 1*A*; see Genetic Manipulation). Haplotype barcoding is ideal for tracking fitness differences between genetic variants in a competitive setting and could be applied to sexually dimorphic variation within and between the sexes. For example, haplotype barcodes could be integrated into multiple genetic backgrounds, crossed to form an ancestral population, and evolved under sex-specific selection or intersexual competition. Unlike traditional E&R experiments which use whole-genome sequencing of the ancestral and evolved population, haplotype barcoding allows for individuals to be sequenced in the smaller barcode at high coverage across many time points throughout evolution. This repeated sequencing provides high-resolution allele frequency traces of different haplotypes to understand the dynamics of genetic variants and how they relate to fitness changes over time. Haplotype barcoding can also be adapted to study differences in recombination rate between the sexes by integrating multiple barcodes at known positions. Differences in recombination rate between the sexes can influence sex-specific genetic architecture (Trivers 1988; Sardell and Kirkpatrick 2020), which could link sex-biased elements contributing to SD. Barcoding at known genomic regions will allow us to follow this process in forward-time experiments and compare recombination rates between the sexes under different environmental conditions.

E&R can also be used to address the relationship between sexually antagonistic selection and SD by generating a negative genetic correlation between female and male fitness (Bonduriansky and Chenoweth 2009) through manipulating sexual selection (Rice 1996; Pitnick, Brown, et al. 2001; Pitnick, Miller, et al. 2001). Again, genetic manipulations introduced before E&R, such as inducible knockdowns or altered expression through CRISPRi (supplementary table 1, Supplementary Material online; box 2), will facilitate understanding the pleiotropic effects of genetic variants in a sex-specific manner. Manipulating expression during E&R can also provide information on how a gene’s interactions are structured within each sex and how these interactions evolve. Additionally, genomic editing can be used to introduce a sex-biased regulatory sequence or genetic variant into the mismatched sex, which will relax the degree of sexual antagonism during E&R and thus reduce the genetic constraints on SD (fig. 1*B* and box 2). Genomic editing also provides the ability to manipulate the sex-determining region and fuse chromosomes together (Shao et al. 2019) to study neo-sex chromosome formation. When used in an E&R framework, this approach will provide insight in real-time on how neo-sex chromosomes evolve and the role of sex chromosomes in resolving sexual conflict (fig. 1*C*).

Despite their power, E&R studies are still sensitive to population size, biological replication, and ancestral haplotype structure (Kofler and Schloetterer 2014; Otte et al. 2020). Importantly, the molecular integration of transgenic elements before E&R homogenizes the genome. To create standing genetic variation for selection to act upon during E&R will require either crossing multiple transgenic strains together or mutagenesis. In the case of crossing, haplotype structure must be carefully considered during experimental design as it can greatly impact the sex-specific response to selection. This approach to E&R relies on manipulating the genome of particular isolates or strains rather than following the genomic response of segregating genetic variants and therefore may not represent all possible evolutionary pathways observed in natural populations. These limitations can be avoided by using transgenics tools only after E&R to verify candidate genes. For example, CRISPR (box 2) can replace a haplotype in the ancestral background with the evolved haplotype (Perli et al. 2020). However, the full benefits of transgenics during E&R will be realized when used as an integrated tool. This goal can be met as transgenics become more efficient and feasible in a range of taxa (supplementary table 1, Supplementary Material online), allowing for multiple strains to be genetically manipulated and crossed.

### Bulked-Segregant Analysis

An alternative approach to E&R is bulked segregant analysis (Brauer et al. 2006), which uses selection on the tails of a phenotypic distribution to map the genetic basis of extreme phenotypes. By repeating over multiple rounds of selection, the variance in the trait can be reduced, which facilitates mapping. Bulked segregant analysis could be a powerful approach to mapping genetic variants of SD and understanding the role of dominance in sexually dimorphic traits, both of which will benefit from existing introgression lines between strains or species. This approach may also be particularly useful for selecting on sex-specific variation during development to map the genetic basis of when and how SD is generated (fig. 1*D*). This approach can be coupled with CRISPR transgenics to validate the function of candidate genes (see Genetic Manipulation). Alternatively, RNA-sequencing and particularly single-cell sequencing can be used to identify expression differences between bulk populations (Ben-David et al. 2020).

### Pedigree Tracing

Pedigree and parent–offspring trio sequencing offer an alternative to E&R for studying sexual dimorphism in populations that are not conducive to experimental evolution, such as in natural populations or organisms with a long generation time (fig. 1*B*; Johnston et al. 2017; Bates et al. 2020; Lucotte et al. 2020). These approaches explicitly correlate haplotype structure with genetic sex and identify recombination events within a population. Additionally, pedigree and trio sequencing approaches explicitly take into account population structure, providing an advantage over genome-wide association studies (GWAS) (Bates et al. 2020). Pedigree tracing has already proved powerful for identifying signatures of selection in wild populations (Johnston et al. 2017; Chen, Juric, et al. 2019). Advances in long-read genomic sequencing and reduced sequencing costs are making these approaches more feasible across taxa. A promising emerging framework being adapted from human genomics is the use of linked-read sequencing to gain insight on phased genomes (Lutgen et al. 2020). Specifically, phased genomic information within a pedigree framework can be used to correlate haplotype structure and local genetic architecture with genetic sex. Additionally, phased genomes gained through linked-sequencing or parent–offspring trio sequencing can be used to study segregation distortion and sexually antagonistic variants within the genome (Lucotte et al. 2020).

### GWAS and High-Resolution Transcriptomics

An alternative approach to manipulating selection is to observe the genomic footprint of selection in natural populations through GWAS or expression association approaches. GWAS provides a powerful approach to associate sex-specific variation in a phenotype with its underlying genetic basis (fig. 1*A*). Taking such a sex-stratified approach will distinguish sex-specific allelic effects (Khramtsova et al. 2019). Although GWAS is sensitive to population demographics, these confounding effects can be controlled for in a logistic regression framework. An association framework also can be used for gene expression data with the potential to reveal how *cis* and *trans* genomic variants influence transcription on a genome-wide scale (Sun and Hu 2013). This framework will be especially powerful when coupled with cell or tissue-specific transcriptomes (see 4D Transcriptomics). The human Genotype-Tissue Expression project (GTEx) is revolutionizing this area of research, identifying over one-third of genes to have a sex-biased expression profile in at least one of the 44 tissues sampled (Oliva et al. 2020). For both data types, sample size will impact the ability to accurately detect signatures of selection and may be a limiting factor for some natural population studies.

Comparative transcriptomic studies provide network-level information about sex-specific node connectivity and redundancy. By coupling classic evolution-development frameworks, particularly during early development, with single-cell sequencing technology (see 4D Transcriptomics), we can begin to create a continuous understanding of SD through time (fig. 1*D*). Spatial transcriptomics has already transformed developmental biology (Farrell et al. 2018; Zhou et al. 2019) and sex-stratified approaches with these methods will only further our knowledge.

### Comparative Genomics and Long-Read Sequencing

Comparative genomic studies focusing on sex chromosomes isolate the genotype space of the genotype–phenotype–fitness map (fig. 1*C*). The relationship between the origin of sex chromosomes and SD is a long-standing area of research (Rice 1984; Charlesworth 1991; Mank 2009; Bachtrog et al. 2011), however, the quality of genome assemblies has been a major limiting factor, especially for nonmodel organisms. Traditional methods for studying sex chromosomes are also benefiting from new technology. Specifically, long-read PacBio and Oxford Nanopore sequencing (Amarasinghe et al. 2020) are providing chromosome length scaffolds for assembling short-read data. These methods generate high-quality assemblies that extend through repetitive regions and tandem duplications, which are problematic for short-read data, but may be common and potentially important components of sex chromosomes (Bachtrog et al. 2019; Peichel et al. 2019; Bracewell and Bachtrog 2020). Similarly, gene duplication and sex-specific functionalization is viewed as an important mechanism leading to the resolution of sexual conflict and the evolution of SD (Gallach et al. 2010; Connallon and Clark 2011; Gallach and Betrán 2011; Wyman et al. 2012). Long-read sequencing can help identify and disentangle recent duplication events more accurately than standard short-read data. Finally, long-read RNA sequencing technologies, such as Iso-seq, are proving to be powerful in identifying sex-specific alternative splicing and the role of this mechanism in the development of sexually dimorphic traits (Zhao et al. 2019).

## Spatial and Temporal Patterns of Sex-Biased Expression and Regulation

Recent advances in sequencing technologies, such as tomographic or spatial transcriptomics (Tomo-seq) and single-cell RNA sequencing (scRNA-seq), allow us to track transcriptomic dynamics across different cell types and tissues, and across development to provide fine-scale resolution of SD in gene expression and regulation. We discuss four methods, which can be used independently or coupled with experimental manipulation to observe the patterns of SD.

### 4D Transcriptomics of SD: scRNA-Seq and Tomo-Seq

Single-cell sequencing expands the feasibility of quantitative gene expression methods across taxa and biological samples. Specifically, single-cell RNA amplification techniques coupled with cell sorting devices offer a major advantage over bulk-cell RNAseq by providing transcript-level expression for thousands or even millions of cells (supplementary table 1, Supplementary Material online; Tang et al. 2009; Islam et al. 2011; Hashimshony et al. 2016; Haque et al. 2017). More recent analytical improvements are enabling postsequencing identification of cell populations by applying advanced clustering and unsupervised learning techniques, such as t-distributed stochastic neighbor embedding (t-SNE), greatly improving the spatial resolution in scRNA-seq data (Kobak and Berens 2019). Although these techniques have largely been used to distinguish gene expression profiles between cell populations within a single individual, scRNA-seq comparisons between the sexes in humans is beginning to unveil the mechanisms of SD (Tukiainen et al. 2017). Additionally, new analytical approaches are enabling differential single-cell expression contrasts between individuals (Butler et al. 2018; Ntranos et al. 2019; Becht et al. 2020), which will facilitate contrasts between cell populations of females and males. Although complexity and expense can build up for an experiment with female and male treatments and multiple developmental time points, a cost-effective, although less high-throughput alternative, is quantitative PCR to monitor pivotal genes on specific cell populations that may have been identified in coarser scans (VanInsberghe et al. 2018). Importantly, scRNA-seq will not only provide an understanding of sex-biased differential expression through development (fig. 1*D*), but can also be used to understand the sex determination cues from sex chromosomes (fig. 1*B*) with spatial and cellular resolution.

Alternatively, Tomo-seq avoids the cell sorting and classification required for scRNA-seq by providing genome-wide gene expression quantification in contiguously cryo-sliced whole-body segments (supplementary table 1, Supplementary Material online; Combs and Eisen 2013; Junker et al. 2014; Kruse et al. 2016). Organisms and developmental stages with low-dimensional bodies, such as embryos, larvae, and worms, are emerging as ideal systems to examine gene expression along anteroposterior, dorso-ventral, and lateral dimensions (Combs and Eisen 2013; Junker et al. 2014; Ebbing et al. 2018). Recent Tomo-seq work in *Caenorhabditis elegans* comparing hermaphrodite and male expression patterns identified the location of genes with sex-biased expression outside of reproductive tissues (Ebbing et al. 2018). Although size remains a limitation for larger-bodied organisms, this technique could be applied, in some cases, to distinguish spatial and functional differences between organs or other low-dimensional structures of females and males (see Wu et al. 2016).

### Measuring Sex-Specific Transcription Binding Activity: ATAC-Seq

Sex-specific regulation can arise in part from transcription factors binding to open chromatin (box 1), yet most of the evidence we have about sex-specific regulation comes from indirect studies of *cis*- and *trans*-regulatory changes in interspecies and interpopulation hybrids (Meiklejohn et al. 2014; Turner et al. 2014; Coolon et al. 2018). To directly address the role of regulation variation in SD, chromatin immunoprecipitation and sequencing (ChIP-seq) and related methods (see Naqvi et al. 2019) can be used to quantify DNA–protein interactions in a high-throughput manner. However, they require a priori knowledge of specific protein targets and large amounts of starting material (Jiang and Mortazavi 2018). Alternatively, the assay for transposase-accessible chromatin sequencing (ATAC-seq, supplementary table 1, Supplementary Material online) is the next iteration of genome-wide DNA–protein interaction assays and overcomes some of these shortcomings by: directly accessing open chromatin enzymatically with the hyperactive Tn5 transposase, not requiring protein-specific markers, allowing for low amounts of starting material, and being time- and cost-efficient (Buenrostro et al. 2013; Yan et al. 2020). Additionally, ATAC-seq is more sensitive, which decreases the signal-to-noise ratio seen in ChIP-seq, and can be integrated into a single-cell sequencing framework (e.g., scATAC-seq). In *C. elegans*, novel regulators have been uncovered using ATAC-seq, revealing complex regulatory dynamics across developmental stages (Daugherty et al. 2017). Overall, ATAC-seq has potential to examine broadly distributed regulatory regions across the genome, which can help disentangle sex-specific binding activity both spatially within the organism and across development.

## Genetic Manipulation for Hypothesis Testing

Many toolkits have been devised to manipulate the genetic architecture and expression of specific genes, allowing for spatiotemporal control and visualization of gene expression to manipulate SD and verify candidate genes. We discuss the feasibility and technical limitations of CRISPR gene engineering (box 2) and highlight four established toolkits, which CRISPR made more accessible.

### Expression Control through Gal4/UAS

The Gal4/UAS system allows for spatiotemporal control of gene expression by splitting the regulation and coding sequence to independently investigate the effects of regulation versus transcription levels on gene function (supplementary table 1, Supplementary Material online). Utilizing sex-specific promoters to drive Gal4 expression allows for feminization or masculinization of specific tissues (fig. 1*B*). For example, sex-specific Gal4 drivers were used to investigate SD in *Drosophila* sleep behaviors (Khericha et al. 2016) and pathology (Regan et al. 2016). Extending these studies to a multigeneration framework will allow for selection to be manipulated in a sex-biased manner to understand the effect of sex-biased regulation on population fitness (fig. 1). Although this system provides a powerful approach to control gene expression, native gene expression is not strictly conserved (see Wang et al. 2017) and must be considered during experimental design and interpretation.

### Expression Control through Cre-Lox

Cre-lox allows for deletion of specific sequences (Gu et al. 1994), translocation of chromosome fragments (Van Deursen et al. 1995), inversion of gene orientation (Grégoire and Kmita 2008), and integration of transgenes (Levy et al. 2015) to manipulate genetic architecture in a controlled manner. Two lox sites are integrated for genetic deletions, flanking the desired sequence to be deleted (supplementary table 1, Supplementary Material online). The expression of Cre induces recombination of the two lox sequences, excising the intermediate stretch of DNA between them. Other utilities simply rely on changing the orientation or location of the lox sites. Under tissue-specific promoters, Cre expression can be controlled spatially and temporally to manipulate sex-specific constraints on SD or alter developmental cues (fig. 1*B* and *D*). Cre-lox has been used to investigate sexually dimorphic behavior and delete the testosterone androgen receptor in mice (Juntti et al. 2008). Although Cre-lox can provide precision control over the desired genetic manipulation, several Cre drivers have transient expression and can lead to the Cre recombinase activity in undesired cells and tissue types (Song and Palmiter 2018). To overcome this obstacle, several “split-Cre” systems can drive portions of the Cre recombinase protein under different drivers, allowing for higher specificity (Hirrlinger et al. 2009).

### Expression Control through Targeted Knockdowns

We can learn about the molecular function underlying SD through controlled and targeted depletion of gene products in both permanent and inducible contexts. Knockdown methodologies, such as RNA interference (RNAi, supplementary table 1, Supplementary Material online) can be used to suppress expression, which provides a powerful tool for examining expression variation between the sexes (Fire et al. 1998). RNAi causes the knockdown of a gene by eliminating the genes’ mRNA by injecting double-strand RNA or in vivo expression (Dzitoyeva et al. 2001; Crotty and Pipkin 2015), and has been adopted in a wide variety of organisms, including humans (Setten et al. 2019). RNAi can be used to verify the necessity and sufficiency of candidate sexually dimorphic genes. For example, RNAi was used to identify the molecular basis of a color SD in the queenless ant, *Diacamma* sp. (Miyazaki et al. 2014), to examine the function of water strider male antennae during mating (Khila et al. 2012), and to test female and male fertility genes in *Drosophila* (Chen et al. 2012; VanKuren and Long 2018; Chen, Delbare, et al. 2019). Although RNAi is a powerful and widely applicable technology across taxa, the effect can be weak and nonspecific degradation can occur (Boutros and Ahringer 2008). In some cases, CRISPRi can overcome these limitations and provide a substitute for RNAi (box 2).

The auxin-inducible degradation (AID, supplementary table 1, Supplementary Material online) system has recently been utilized for targeted gene knockdown (Nishimura et al. 2009). AID uses a transgenic plant protein, TIR1, which recognizes a small specific degron tag on a protein of interest and degrades this protein in the presence of auxin. The degron tag can be added to native genes by CRISPR (box 2), or transgenic integrations of genes with the degron tag can be introduced into a wild-type or mutant background. Importantly, AID has higher specificity compared with RNAi and temporal control is simpler to achieve through the addition of auxin. Despite its power, AID is sensitive to the concentration of auxin, less permeable in some tissues, and auxin-independent degradation has been observed (Zhang et al. 2015; Papagiannakis et al. 2017; Schiksnis et al. 2020). AID has successfully been used for protein depletion in cell culture and animal models (Kanke et al. 2011; Holland et al. 2012; Zhang et al. 2015; Kasimatis et al. 2018), except in zebrafish where the current form of the AID system has a limited effect (Yamaguchi et al. 2019). To the best of our knowledge, AID has not been specifically applied to questions of SD, however, this method is ideal for manipulating sex-limited selection in an E&R framework (fig. 1*A*).

### Haplotype Tracking through Fluorescent Reporters and Barcoding

The ability to visually mark when and where a gene gets expressed is arguably the most basic and essential tool utilized by molecular genetics to investigate genetic architecture and can provide a visual context for expression differences between the sexes. Fluorescent reporters can be tagged to a native protein or act as an independent transgene (box 2) and have been developed in many color variants for a wide range of utilities (Rodriguez et al. 2017), including competition experiments to identify adaptive lineages (Hegreness et al. 2006; Crombie et al. 2018) and sex-stratified experiments to parse sexually dimorphic gene expression (Serrano-Saiz et al. 2017). However, some fluorescent reporters are very dim depending on the transcriptional activity, and translational reporters, in some cases, can disrupt protein activity, which prevents the incorporation of a fluorescent tag.

Although fluorescent reporters allow for simple identification, the total number of reporters are significantly limited. High-throughput approaches that include neutral genomic-integrations—namely barcodes—have recently been adapted to study adaptive lineages in yeast and bacteria (Blundell and Levy 2014; Levy et al. 2015; Jasinska et al. 2020). Although barcoded lineage-tracking has not been explicitly adopted in animal systems, unique lineage identification has been implemented in competitive experiments utilizing reporters (Marie-Orleach et al. 2016). Expanding on fluorescent reporter marked lineages, various sex-specific lineages could be created and marked for competition experiments (fig. 1*A*). After overcoming the technical limitation of genomic barcoding, high-throughput lineage tracking will be the next great breakthrough in experimental evolution in animal systems.

## Conclusions

SD constitutes much of the diversity observed between organisms and is integrated across the genotype–phenotype–fitness map. By harnessing cutting-edge methods developed for molecular biology and biomedical research, we can design explicit experiments to address how this remarkable diversity evolved from a shared genome. With few exceptions, the technological advancements discussed here will allow us to increase the spatial, temporal, and molecular resolution of the underpinnings of SD, and expand our ability to implement molecular and genetic studies in nonmodel organisms. The field is poised to synergize advances in molecular biology and sequencing technology within evolutionary frameworks, promising novel insights on the creation and maintenance of SD in the near future.

## Supplementary Material

Supplementary data are available at *Genome Biology and Evolution* online.

Box 1.—Molecular Mechanisms That Can Contribute to Sex Differences.

**Figure:**
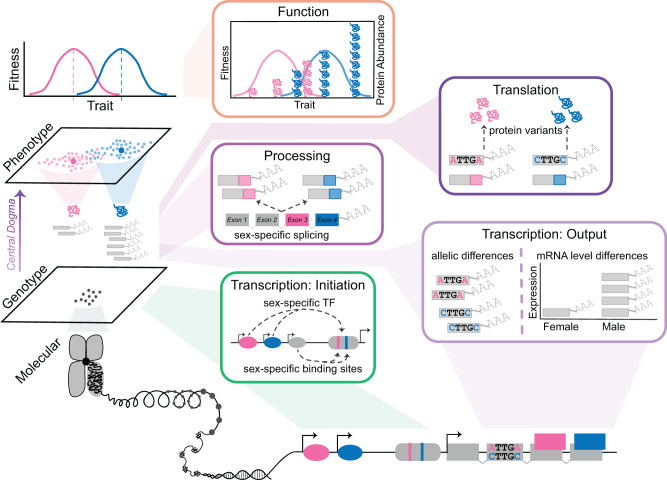

To understand how sex-specific selection drives the evolution of traits in populations, we need some understanding of the underlying genetic basis of the traits, as highlighted in Lewontin’s (1974) classic text. A critical component of Lewontin’s genotype–phenotype map is the first transformation, which encompasses the central dogma of molecular biology: DNA to RNA to protein (as shown below). These molecular underpinnings are particularly important in the context of SD as the largely, and in some cases completely, shared genetic material is producing distinct phenotypes within each sex.

Phenotypic variation can arise through modifications to coding sequences including gene duplication, changes in gene regulation, and modifications during translation (King and Wilson 1975; Levine and Tjian 2003; Wyman et al. 2012; Grath and Parsch 2016; Mank 2017; Khramtsova et al. 2019). Sex differences can be generated by completely sex-limited genes, often located on sex chromosomes (Mank 2009) or by genetically encoded differences in the initiation of transcription (shown in green). Here, differences in transcription factor (TF) binding frequency between the sexes or sex-specific TF binding sites will drive sex-specific mRNA levels. Additionally, sex-biased deployment of master regulators can initiate a cascade of sexual differentiation.

Transcription can be divided into two stages: the production of mRNA transcripts followed by processing of these transcripts (shown in purple). Differences in transcriptional output between the sexes include overall differences in mRNA expression levels generated through either differential TF binding or sex-specific degradation of mRNA. Alternatively, a sexually antagonistic polymorphism can generate allelic differences in mRNA transcripts between the sexes. Ultimately, this effect is not realized unless the translated protein variants differ in form and function between the sexes (shown in dark purple). During the posttranscriptional regulation stage, sex-specific alternative splicing (Chang et al. 2011; Hartmann et al. 2011; Li et al. 2013) and small RNA regulators (Warnefors et al. 2017; Bezler et al. 2019) generate sex-specific mRNA (as shown in purple). As with a sexually antagonistic polymorphism, this effect is only realized if the sex-specific isoforms have protein variants that differ in form or function (shown in orange).

Although not exhaustive, this list includes the major mechanisms that have been identified or hypothesized to contribute to sexual dimorphism. However, more work is needed to determine if one mechanism is more common than others or if the molecular mechanisms contributing to SD differ for simple versus complex traits.

Box 2.—Genetic Manipulation through CRISPR/Cas.

**Figure:**
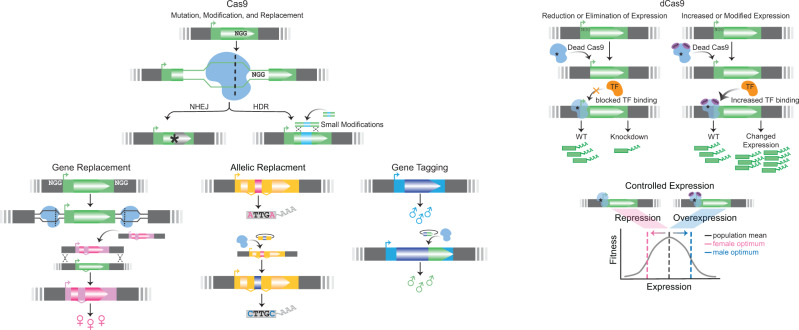

CRISPR (clustered regularly interspaced short palindromic repeats) has become the premier method for mutating and editing the genome with precision (Jinek et al. 2012; Doudna and Charpentier 2014; Pickar-Oliver and Gersbach 2019). Although the vast scope of CRISPR utilities cannot be represented here, we outline three mechanisms and their application to SD. Cas9, the most common nuclease associated with the CRISPR system, is a targetable nuclease, which provides experimental control over the location of the nuclease activity. Cas9 and other CRISPR-associated nucleases are “guided” by specific single-stranded RNA encoding the sequence of interest. The PAM (Protospacer Adjacent Motif) sequence restricts the locations of Cas9 targeting. Cas9 targets guide site locations with “NGG” PAM sequence and cleaves double-strand DNA (shown above). When Cas9 creates a break, the cell will attempt to repair the break by nonhomologous end joining (NHEJ) or homology directed repair (HDR). NHEJ is error-prone and thus a simple way to create mutations in the desired gene. However, depending on the mutation’s location, the protein may still fold and function properly. HDR modifies a gene in a specific manner through a “donor template” to repair the DSB (reviewed in Doudna and Charpentier 2014). This process can use the homologous chromosome as a template or non-native DNA template sequence can be introduced to the genome. The synthetic construct (e.g., plasmid, PCR product, or single-stranded oligo) must contain sequences homologous to the genome on both the 5′ and 3′ sides of the desired insert to co-opt the genome repair machinery for integration. HDR has many applications in the study of SD (as shown above). For example, whole genes can be deleted to determine the gene’s sex-specific function. Alternatively, whole genes or promoter regions can be replaced with a sex-biased version to test sex-specific function. A more focused approach would directly edit alleles to verify the significance of sexually antagonistic polymorphisms. Beyond verifying the function of genetic elements in generating SD, HDR can also be used to add epitopes or fluorescent tags to aid during experimentation (see fig. 1 and Genetic Manipulations).

Catalytically inactive variants of Cas9, dead Cas9 (dCas9), target and bind to the sequence without cutting the genome (shown above). In doing so, the dCas9 protein physically blocks the binding of transcription factors and creates a knock-down phenotype (Larson et al. 2016; Pickar-Oliver and Gersbach 2019). This utility, termed CRISPRi, is analogous to RNAi, and may complement the knockdown phenotype or be an adequate replacement (Stojic et al. 2018). Alternatively, dCas9 can be fused to various transcription activators, such as VP64, to change the target genes’ expression level and specificity (reviewed in Pickar-Oliver and Gersbach 2019). By changing the regulatory sequences driving dCas9 expression, specific genes can be experimentally altered in a tissue-specific or cell-specific manner. Experimentally, this manipulation of expression opens many possibilities to directly manipulate genetic architecture in a sex-biased way.

Although CRISPR’s successes have been widespread (supplementary table 1, Supplementary Material online), unique challenges exist for species-specific utility. Targeting the genome requires knowing the DNA sequence and the genomic location of sequences. Adapting CRISPR for new species or highly divergent strains may be complicated if this information is lacking. Additionally, gene duplications and pseudogenes which arose from duplications can also pose a challenge since they likely share much of their sequence in common. Again, high-quality genome assemblies can help to control for this problem, though off-target CRISPR effects can still occur. Finally, delivery of Cas9 and the guide RNA is unique to each species and will require optimizing the method with that species to achieve targeted genome-editing. Nevertheless, CRISPR has been widely adopted among may species and nonmodels with great success; recent protocols developed for nonmodel organisms, including firebug *Pyrrhocoris apterus* (Kotwica-Rolinska et al. 2019), malaria mosquitos (Hammond et al. 2017), and lizards (Rasys et al. 2019). This continued methodological progress, coupled with advances in sequencing technology, will expand the potential applications of CRISPR across taxa.

## Supplementary Material

evaa243_Supplementary_Data

## References

[evaa243-B1] Amarasinghe SL , et al 2020 Opportunities and challenges in long-read sequencing data analysis. Genome Biol. 21(1):30–16. 32033565 10.1186/s13059-020-1935-5 PMC7006217

[evaa243-B2] Arnqvist G , Rowe L. 2005 Sexual conflict. Princeton (NY): Princeton University Press.

[evaa243-B3] Bachtrog D , et al 2011 Are all sex chromosomes created equal? Trends Genet. 27(9):350–357. 21962970 10.1016/j.tig.2011.05.005

[evaa243-B4] Bachtrog D , Mahajan S, Bracewell R. 2019 Massive gene amplification on a recently formed Drosophila Y chromosome. Nat Ecol Evol. 3(11):1587–1597. 31666742 10.1038/s41559-019-1009-9 PMC7217032

[evaa243-B5] Barrett RDH , Hoekstra HE. 2011 Molecular spandrels: tests of adaptation at the genetic level. Nat Rev Genet. 12(11):767–780. 22005986 10.1038/nrg3015

[evaa243-B6] Bates S , Sesia M, Sabatti C, Candès E. 2020 Causal inference in genetic trio studies. Proc Natl Acad Sci U S A. 52:1–10. 10.1073/pnas.2007743117 PMC7533659 32948695

[evaa243-B7] Becht E , Zhao E, Amezquita R, Gottardo R. 2020 Aggregating transcript-level analyses for single-cell differential gene expression. Nat Methods. 17(6):583–585. 32483334 10.1038/s41592-020-0854-4

[evaa243-B8] Ben-David E , et al 2020 Whole-organism eQTL mapping at cellular resolution with single-cell sequencing. bioRxiv. 1–45. 10.1101/2020.08.23.263798. PMC8062134 33734084

[evaa243-B9] Bezler A , et al 2019 Tissue- and sex-specific small RNAomes reveal sex differences in response to the environment. PLoS Genet. 15(2):e1007905. 30735500 10.1371/journal.pgen.1007905 PMC6383947

[evaa243-B10] Blundell JR , Levy SF. 2014 Beyond genome sequencing: lineage tracking with barcodes to study the dynamics of evolution, infection, and cancer. Genomics 104(6):417–430. 25260907 10.1016/j.ygeno.2014.09.005

[evaa243-B11] Bohne A , Sengstag T, Salzburger W. 2014 Comparative transcriptomics in East African cichlids reveals sex- and species-specific expression and new candidates for sex differentiation in fishes. Genome Biol Evol. 6(9):2567–2585. 25364805 10.1093/gbe/evu200 PMC4202336

[evaa243-B12] Bonduriansky R , Chenoweth SF. 2009 Intralocus sexual conflict. Trends Ecol Evol. 24:1–9. 19307043 10.1016/j.tree.2008.12.005

[evaa243-B13] Boutros M , Ahringer J. 2008 The art and design of genetic screens: RNA interference. Nat Rev Genet. 9(7):554–566. 18521077 10.1038/nrg2364

[evaa243-B14] Bracewell R , Bachtrog D. 2020 Complex evolutionary history of the Y chromosome in flies of the *Drosophila obscura* species group. Genome Biol Evol. 12(5):494–505. 32176296 10.1093/gbe/evaa051 PMC7199386

[evaa243-B15] Brauer MJ , Christianson CM, Pai DA, Dunham MJ. 2006 Mapping novel traits by array-assisted bulk segregant analysis in *Saccharomyces cerevisiae*. Genetics 173(3):1813–1816. 16624899 10.1534/genetics.106.057927 PMC1526703

[evaa243-B16] Buenrostro JD , Giresi PG, Zaba LC, Chang HY, Greenleaf WJ. 2013 Transposition of native chromatin for fast and sensitive epigenomic profiling of open chromatin, DNA-binding proteins and nucleosome position. Nat Methods. 10(12):1213–1218. 24097267 10.1038/nmeth.2688 PMC3959825

[evaa243-B17] Butler A , Hoffman P, Smibert P, Papalexi E, Satija R. 2018 Integrating single-cell transcriptomic data across different conditions, technologies, and species. Nat Biotechnol. 36(5):411–420. 29608179 10.1038/nbt.4096 PMC6700744

[evaa243-B18] Chang PL , Dunham JP, Nuzhdin SV, Arbeitman MN. 2011 Somatic sex-specific transcriptome differences in *Drosophila* revealed by whole transcriptome sequencing. BMC Genomics 12(1):364–420. 21756339 10.1186/1471-2164-12-364 PMC3152543

[evaa243-B19] Charlesworth B. 1991 The evolution of sex chromosomes. Science 251(4997):1030–1033. 1998119 10.1126/science.1998119

[evaa243-B20] Chen DS , Delbare SYN, et al 2019 Female genetic contributions to sperm competition in *Drosophila melanogaster*. Genetics 212(3):789–800. 31101677 10.1534/genetics.119.302284 PMC6614900

[evaa243-B21] Chen N , Juric I, et al 2019 Allele frequency dynamics in a pedigreed natural population. Proc Natl Acad Sci U S A. 116(6):2158–2164. 30598449 10.1073/pnas.1813852116 PMC6369762

[evaa243-B22] Chen S , et al 2012 Reshaping of global gene expression networks and sex-biased gene expression by integration of a young gene. EMBO J. 31(12):2798–2809. 22543869 10.1038/emboj.2012.108 PMC3380208

[evaa243-B23] Chenoweth SF , Rundle HD, Blows MW. 2008 Genetic constraints and the evolution of display trait sexual dimorphism by natural and sexual selection. Am Nat. 171(1):22–34. 18171148 10.1086/523946

[evaa243-B24] Combs PA , Eisen MB. 2013 Sequencing mRNA from cryo-sliced *Drosophila* embryos to determine genome-wide spatial patterns of gene expression. PLoS One 8(8):e71820. 23951250 10.1371/journal.pone.0071820 PMC3741199

[evaa243-B25] Connallon T , Clark AG. 2011 The resolution of sexual antagonism by gene duplication. Genetics 187(3):919–937. 21220356 10.1534/genetics.110.123729 PMC3063682

[evaa243-B26] Coolon JD , McManus CJ, Stevenson KR, Graveley BR, Wittkopp PJ. 2018 Corrigendum: tempo and mode of regulatory evolution in *Drosophila*. Genome Res. 28(11):1766–1766. 30385616 10.1101/gr.244087.118 PMC6211639

[evaa243-B27] Crombie TA , Saber S, Saxena AS, Egan R, Baer CF. 2018 Head-to-head comparison of three experimental methods of quantifying competitive fitness in *C. elegans*. PLoS One 13(10):e0201507. 30339672 10.1371/journal.pone.0201507 PMC6195253

[evaa243-B28] Crotty S , Pipkin ME. 2015 In vivo RNAi screens: concepts and applications. Trends Immunol. 36(5):315–322. 25937561 10.1016/j.it.2015.03.007 PMC4810674

[evaa243-B29] Cutter AD , Ward S. 2005 Sexual and temporal dynamics of molecular evolution in *C. elegans* development. Mol Biol Evol. 22(1):178–188. 15371532 10.1093/molbev/msh267

[evaa243-B30] Daugherty AC , et al 2017 Chromatin accessibility dynamics reveal novel functional enhancers in *C. elegans*. Genome Res. 27(12):2096–2107. 29141961 10.1101/gr.226233.117 PMC5741055

[evaa243-B31] Doudna JA , Charpentier E. 2014 Genome editing. The new frontier of genome engineering with CRISPR-Cas9. Science 346(6213):1258096. 25430774 10.1126/science.1258096

[evaa243-B32] Douglas C , et al 2020 Generating single sex litters: development of CRISPR-Cas9 genetic tools to produce all-male offspring. bioRxiv. 1–23. 10.1101/2020.09.07.285536.

[evaa243-B33] Dzitoyeva S , Dimitrijevic N, Manev H. 2001 Intra-abdominal injection of double-stranded RNA into anesthetized adult *Drosophila* triggers RNA interference in the central nervous system. Mol Psychiatry. 6(6):665–670. 11673794 10.1038/sj.mp.4000955

[evaa243-B34] Ebbing A , et al 2018 Spatial transcriptomics of *C. elegans* males and hermaphrodites identifies sex-specific differences in gene expression patterns. Dev Cell. 47(6):801–813.e6. 30416013 10.1016/j.devcel.2018.10.016

[evaa243-B35] Edward DA , Fricke C, Chapman T. 2010 Adaptations to sexual selection and sexual conflict: insights from experimental evolution and artificial selection. Philos Trans R Soc B. 365(1552):2541–2548. 10.1098/rstb.2010.0027 PMC2935098 20643744

[evaa243-B36] Farrell JA , et al 2018 Single-cell reconstruction of developmental trajectories during zebrafish embryogenesis. Science 360(6392):eaar3131. 29700225 10.1126/science.aar3131 PMC6247916

[evaa243-B37] Fire A , et al 1998 Potent and specific genetic interference by double-stranded RNA in *Caenorhabditis elegans*. Nature 391(6669):806–811. 9486653 10.1038/35888

[evaa243-B38] Gallach M , Betrán E. 2011 Intralocus sexual conflict resolved through gene duplication. Trends Ecol Evol. 26(5):222–228. 21397976 10.1016/j.tree.2011.02.004 PMC3090214

[evaa243-B39] Gallach M , Chandrasekaran C, Betrán E. 2010 Analyses of nuclearly encoded mitochondrial genes suggest gene duplication as a mechanism for resolving intralocus sexually antagonistic conflict in *Drosophila*. Genome Biol Evol. 2:835–850. 21037198 10.1093/gbe/evq069 PMC2995371

[evaa243-B40] Grath S , Parsch J. 2016 Sex-biased gene expression. Annu Rev Genet. 50(1):29–44. 27574843 10.1146/annurev-genet-120215-035429

[evaa243-B41] Grégoire D , Kmita M. 2008 Recombination between inverted loxP sites is cytotoxic for proliferating cells and provides a simple tool for conditional cell ablation. Proc Natl Acad Sci U S A. 105(38):14492–14496. 18787116 10.1073/pnas.0807484105 PMC2567143

[evaa243-B42] Gu H , Marth JD, Orban PC, Mossmann H, Rajewsky K. 1994 Deletion of a DNA polymerase beta gene segment in T cells using cell type-specific gene targeting. Science 265(5168):103–106. 8016642 10.1126/science.8016642

[evaa243-B43] Hammond AM , et al 2017 The creation and selection of mutations resistant to a gene drive over multiple generations in the malaria mosquito. PLoS Genet. 13(10):e1007039. 28976972 10.1371/journal.pgen.1007039 PMC5648257

[evaa243-B44] Haque A , Engel J, Teichmann SA, Lönnberg T. 2017 A practical guide to single-cell RNA-sequencing for biomedical research and clinical applications. Genome Med. 9(1):75–12. 28821273 10.1186/s13073-017-0467-4 PMC5561556

[evaa243-B45] Harrison PW , et al 2015 Sexual selection drives evolution and rapid turnover of male gene expression. Proc Natl Acad Sci U S A. 112(14):4393–4398. 25831521 10.1073/pnas.1501339112 PMC4394296

[evaa243-B46] Hartmann B , et al 2011 Distinct regulatory programs establish widespread sex-specific alternative splicing in *Drosophila melanogaster*. RNA 17(3):453–468. 21233220 10.1261/rna.2460411 PMC3039145

[evaa243-B47] Hashimshony T , et al 2016 CEL-Seq2: sensitive highly-multiplexed single-cell RNA-Seq. Genome Biol. 17(1):77–77. 27121950 10.1186/s13059-016-0938-8 PMC4848782

[evaa243-B48] Hegreness M , Shoresh N, Hartl D, Kishony R. 2006 An equivalence principle for the incorporation of favorable mutations in asexual populations. Science 311(5767):1615–1617. 16543462 10.1126/science.1122469

[evaa243-B49] Hirrlinger J , et al 2009 Split-cre complementation indicates coincident activity of different genes in vivo. PLoS One 4(1):e4286. 19172189 10.1371/journal.pone.0004286 PMC2628726

[evaa243-B50] Holland AJ , Fachinetti D, Han JS, Cleveland DW. 2012 Inducible, reversible system for the rapid and complete degradation of proteins in mammalian cells. Proc Natl Acad Sci U S A. 109(49):E3350–E3357. 23150568 10.1073/pnas.1216880109 PMC3523849

[evaa243-B51] Hsu S-K , et al 2020 Rapid sex-specific adaptation to high temperature in *Drosophila*. eLife 9:1083–1016. 10.7554/eLife.53237 PMC7034977 32083552

[evaa243-B52] Innocenti P , Morrow EH. 2010 The sexually antagonistic genes of *Drosophila melanogaster*. PLoS Biol. 8(3):e1000335–10. 20305719 10.1371/journal.pbio.1000335 PMC2838750

[evaa243-B53] Islam S , et al 2011 Characterization of the single-cell transcriptional landscape by highly multiplex RNA-seq. Genome Res. 21(7):1160–1167. 21543516 10.1101/gr.110882.110 PMC3129258

[evaa243-B54] Jasinska W , et al 2020 Chromosomal barcoding of E. coli populations reveals lineage diversity dynamics at high resolution. Nat Ecol Evol. 4(3):437–452. 32094541 10.1038/s41559-020-1103-z

[evaa243-B55] Jiang S , Mortazavi A. 2018 Integrating ChIP-seq with other functional genomics data. Brief Funct Genomics. 17(2):104–115. 29579165 10.1093/bfgp/ely002 PMC5888983

[evaa243-B56] Jin W , et al 2001 The contributions of sex, genotype and age to transcriptional variance in *Drosophila melanogaster*. Nat Genet. 29(4):389–395. 11726925 10.1038/ng766

[evaa243-B57] Jinek M , et al 2012 A programmable dual-RNA-guided DNA endonuclease in adaptive bacterial immunity. Science 337(6096):816–821. 22745249 10.1126/science.1225829 PMC6286148

[evaa243-B58] Johnston SE , Huisman J, Ellis PA, Pemberton JM. 2017 A high-density linkage map reveals sexual dimorphism in recombination landscapes in red deer (*Cervus elaphus*). G3 (Bethesda) 7:2859–2870. 28667018 10.1534/g3.117.044198 PMC5555489

[evaa243-B59] Junker JP , et al 2014 Genome-wide RNA Tomography in the zebrafish embryo. Cell 159(3):662–675. 25417113 10.1016/j.cell.2014.09.038

[evaa243-B60] Juntti SA , Coats JK, Shah NM. 2008 A genetic approach to dissect sexually dimorphic behaviors. Horm Behav. 53(5):627–637. 18313055 10.1016/j.yhbeh.2007.12.012 PMC2464277

[evaa243-B61] Kanke M , et al 2011 Auxin-inducible protein depletion system in fission yeast. BMC Cell Biol. 12(1):8. 21314938 10.1186/1471-2121-12-8 PMC3048574

[evaa243-B62] Kasimatis KR , Moerdyk-Schauwecker MJ, Phillips PC. 2018 Auxin-mediated sterility induction system for longevity and mating studies in *Caenorhabditis elegans*. G3 (Bethesda) 8:2655–2662. 29880556 10.1534/g3.118.200278 PMC6071612

[evaa243-B63] Khericha M , Kolenchery JB, Tauber E. 2016 Neural and non-neural contributions to sexual dimorphism of mid-day sleep in *Drosophila melanogaster*: a pilot study. Physiol Entomol. 41(4):327–334. 27840547 10.1111/phen.12134 PMC5091642

[evaa243-B64] Khila A , Abouheif E, Rowe L. 2012 Function, developmental genetics, and fitness consequences of a sexually antagonistic trait. Science 336(6081):585–589. 22556252 10.1126/science.1217258

[evaa243-B65] Khramtsova EA , Davis LK, Stranger BE. 2019 The role of sex in the genomics of human complex traits. Nat Rev Genet. 20(3):173–190. 30581192 10.1038/s41576-018-0083-1

[evaa243-B66] King MC , Wilson AC. 1975 Evolution at two levels in humans and chimpanzees. Science 188(4184):107–116. 1090005 10.1126/science.1090005

[evaa243-B67] Kobak D , Berens P. 2019 The art of using t-SNE for single-cell transcriptomics. Nat Commun. 10(1):5416–5414. 31780648 10.1038/s41467-019-13056-x PMC6882829

[evaa243-B68] Kofler R , Schloetterer C. 2014 A guide for the design of evolve and resequencing studies. Mol Biol Evol. 31(2):474–483. 24214537 10.1093/molbev/mst221 PMC3907048

[evaa243-B69] Kotwica-Rolinska J , et al 2019 CRISPR/Cas9 genome editing introduction and optimization in the non-model insect *Pyrrhocoris apterus*. Front Physiol. 10:1–15. 31379599 10.3389/fphys.2019.00891 PMC6644776

[evaa243-B70] Kruse F , Junker JP, van Oudenaarden A, Bakkers J. 2016 Tomo-seq: a method to obtain genome-wide expression data with spatial resolution. Methods Cell Biol. 135:299–307. 27443932 10.1016/bs.mcb.2016.01.006

[evaa243-B71] Larson EL , et al 2016 Contrasting levels of molecular evolution on the mouse X chromosome. Genetics 203(4):1841–1857. 27317678 10.1534/genetics.116.186825 PMC4981281

[evaa243-B72] Levine M , Tjian R. 2003 Transcription regulation and animal diversity. Nature 424(6945):147–151. 12853946 10.1038/nature01763

[evaa243-B73] Levy SF , et al 2015 Quantitative evolutionary dynamics using high-resolution lineage tracking. Nature 519(7542):181–186. 25731169 10.1038/nature14279 PMC4426284

[evaa243-B74] Lewontin RC. 1974 The genetic basis of evolutionary change. New York: Columbia University Press.

[evaa243-B75] Li F , Vensko SP, Belikoff EJ, Scott MJ. 2013 Conservation and sex-specific splicing of the transformer gene in the calliphorids *Cochliomyia hominivorax, Cochliomyia macellaria and Lucilia sericata*. PLoS One 8(2):e56303. 23409170 10.1371/journal.pone.0056303 PMC3567074

[evaa243-B76] Lucotte EA , et al 2020 Detection of sexually antagonistic transmission distortions in trio datasets. bioRxiv. 1–21. 10.1101/2020.09.11.293191. PMC8966469 35386833

[evaa243-B77] Lutgen D , et al 2020 Linked-read sequencing enables haplotype-resolved resequencing at population scale. Mol Ecol Resour. 20(5):1311–1322. 32419391 10.1111/1755-0998.13192

[evaa243-B78] Maklakov AA , Bonduriansky R, Brooks RC. 2009 Sex differences, sexual selection, and ageing: an experimental evolution approach. Evolution 63(10):2491–2503. 19519633 10.1111/j.1558-5646.2009.00750.x

[evaa243-B79] Mank JE. 2009 Sex chromosomes and the evolution of sexual dimorphism: lessons from the genome. Am Nat. 173(2):141–150. 20374139 10.1086/595754

[evaa243-B80] Mank JE. 2017 The transcriptional architecture of phenotypic dimorphism. Nat Rev Genet. 1:1–7. 10.1038/s41559-016-0006 28812569

[evaa243-B81] Marie-Orleach L , Janicke T, Vizoso DB, David P, Schärer L. 2016 Quantifying episodes of sexual selection: insights from a transparent worm with fluorescent sperm. Evolution 70(2):314–328. 26787006 10.1111/evo.12861

[evaa243-B82] Meiklejohn CD , Coolon JD, Hartl DL, Wittkopp PJ. 2014 The roles of cis- and trans-regulation in the evolution of regulatory incompatibilities and sexually dimorphic gene expression. Genome Res. 24(1):84–95. 24043293 10.1101/gr.156414.113 PMC3875864

[evaa243-B83] Miyazaki S , et al 2014 Sexually dimorphic body color is regulated by sex-specific expression of yellow gene in ponerine ant, *Diacamma* sp. PLoS One 9(3):e92875. 24667821 10.1371/journal.pone.0092875 PMC3965500

[evaa243-B84] Naqvi S , et al 2019 Conservation, acquisition, and functional impact of sex-biased gene expression in mammals. Science 365(6450):eaaw7317–10. 31320509 10.1126/science.aaw7317 PMC6896219

[evaa243-B85] Nishimura K , Fukagawa T, Takisawa H, Kakimoto T, Kanemaki M. 2009 An auxin-based degron system for the rapid depletion of proteins in nonplant cells. Nat Methods. 6(12):917–922. 19915560 10.1038/nmeth.1401

[evaa243-B86] Ntranos V , Yi L, Melsted P, Pachter L. 2019 A discriminative learning approach to differential expression analysis for single-cell RNA-seq. Nat Methods. 16(2):163–166. 30664774 10.1038/s41592-018-0303-9

[evaa243-B87] Oliva M , et al 2020 The impact of sex on gene expression across human tissues. Science 369(6509):eaba3066–eaba3116. 32913072 10.1126/science.aba3066 PMC8136152

[evaa243-B88] Otte KA , Nolte V, Mallard F, Schlötterer C. 2020 The adaptive architecture is shaped by population ancestry and not by selection regime. bioRxiv. 1–38. 10.1101/2020.06.25.170878. PMC8285869 34271951

[evaa243-B89] Papagiannakis A , de Jonge JJ, Zhang Z, Heinemann M. 2017 Quantitative characterization of the auxin-inducible degron: a guide for dynamic protein depletion in single yeast cells. Sci Rep. 7(1):1–13. 28680098 10.1038/s41598-017-04791-6 PMC5498663

[evaa243-B90] Parker G. 1979 Sexual selection and sexual conflict. New York: Academic Press.

[evaa243-B91] Peichel CL , et al 2019 Assembly of a young vertebrate Y chromosome reveals convergent signatures of sex chromosome evolution. bioRxiv. 1–47. 10.1101/2019.12.12.874701. PMC7368989 32684159

[evaa243-B92] Perli T , Moonen DPI, van den Broek M, Pronk JT, Daran J-M. 2020 Adaptive laboratory evolution and reverse engineering of single-vitamin prototrophies in *Saccharomyces cerevisiae*. Appl Environ Microbiol. 86(12):965–923. 10.1128/AEM.00388-20 PMC7267190 32303542

[evaa243-B93] Pickar-Oliver A , Gersbach CA. 2019 The next generation of CRISPR-Cas technologies and applications. Nat Rev Mol Cell Biol. 20(8):490–507. 31147612 10.1038/s41580-019-0131-5 PMC7079207

[evaa243-B94] Pitnick S , Brown WD, Miller GT. 2001 Evolution of female remating behaviour following experimental removal of sexual selection. Proc R Soc Lond B. 268(1467):557–563. 10.1098/rspb.2000.1400 PMC1088640 11297171

[evaa243-B95] Pitnick S , Miller GT, Reagan J, Holland B. 2001 Males’ evolutionary responses to experimental removal of sexual selection. Proc R Soc Lond B. 268(1471):1071–1089. 10.1098/rspb.2001.1621 PMC1088710 11375092

[evaa243-B96] Ranz JM , Castillo-Davis CI, Meiklejohn CD, Hartl DL. 2003 Sex-dependent gene expression and evolution of the *Drosophila* transcriptome. Science 300(5626):1742–1745. 12805547 10.1126/science.1085881

[evaa243-B97] Rasys AM , et al 2019 CRISPR-Cas9 gene editing in lizards through microinjection of unfertilized oocytes. Cell Rep. 28(9):2288–2292.e3. 31461646 10.1016/j.celrep.2019.07.089 PMC6727204

[evaa243-B98] Regan JC , et al 2016 Sex difference in pathology of the ageing gut mediates the greater response of female lifespan to dietary restriction. eLife 5:75. 10.7554/eLife.10956 PMC4805549 26878754

[evaa243-B99] Reinke V , et al 2000 A global profile of germline gene expression in *C. elegans*. Mol Cell. 6(3):605–616. 11030340 10.1016/s1097-2765(00)00059-9

[evaa243-B100] Rice WR. 1984 Sex chromosomes and the evolution of sexual dimorphism. Evolution 38(4):735–742. 28555827 10.1111/j.1558-5646.1984.tb00346.x

[evaa243-B101] Rice WR. 1996 Sexually antagonistic male adaptation triggered by experimental arrest of female evolution. Nature 381(6579):232–234. 8622764 10.1038/381232a0

[evaa243-B102] Rodriguez EA , et al 2017 The growing and glowing toolbox of fluorescent and photoactive proteins. Trends Biochem Sci. 42(2):111–129. 27814948 10.1016/j.tibs.2016.09.010 PMC5272834

[evaa243-B103] Rostant WG , Mason JS, de Coriolis J-C, Chapman T. 2020 Resource-dependent evolution of female resistance responses to sexual conflict. Evol Lett. 4(1):54–64. 32055411 10.1002/evl3.153 PMC7006461

[evaa243-B104] Sardell JM , Kirkpatrick M. 2020 Sex differences in the recombination landscape. Am Nat. 195(2):361–379. 32017625 10.1086/704943 PMC7537610

[evaa243-B105] Schiksnis E , et al 2020 Auxin-independent depletion of degron-tagged proteins by TIR1. MicroPubl Biol. 2020. doi:10.17912/micropub.biology.000213. 10.17912/micropub.biology.000213 PMC7252403 32550513

[evaa243-B106] Schlötterer C , Kofler R, Versace E, Tobler R, Franssen SU. 2015 Combining experimental evolution with next-generation sequencing: a powerful tool to study adaptation from standing genetic variation. Heredity 114(5):431–440. 25269380 10.1038/hdy.2014.86 PMC4815507

[evaa243-B107] Serrano-Saiz E , Oren-Suissa M, Bayer EA, Hobert O. 2017 Sexually dimorphic differentiation of a *C. elegans* hub neuron is cell autonomously controlled by a conserved transcription factor. Curr Biol. 27(2):199–209. 28065609 10.1016/j.cub.2016.11.045 PMC5805387

[evaa243-B108] Setten RL , Rossi JJ, Han S-P. 2019 The current state and future directions of RNAi-based therapeutics. Nat Rev Drug Discov. 18(6):421–446. 30846871 10.1038/s41573-019-0017-4

[evaa243-B109] Shao Y , Lu N, Xue X, Qin Z. 2019 Creating functional chromosome fusions in yeast with CRISPR-Cas9. Nat Protoc. 14(8):2521–2545. 31300803 10.1038/s41596-019-0192-0

[evaa243-B110] Snook RR , Gidaszewski NA, Chapman T, Simmons LW. 2013 Sexual selection and the evolution of secondary sexual traits: sex comb evolution in *Drosophila*. J Evol Biol. 26(4):912–918. 23496332 10.1111/jeb.12105

[evaa243-B111] Song AJ , Palmiter RD. 2018 Detecting and avoiding problems when using the Cre-lox system. Trends Genet. 34(5):333–340. 29336844 10.1016/j.tig.2017.12.008 PMC5910175

[evaa243-B112] Stojic L , et al 2018 Specificity of RNAi, LNA and CRISPRi as loss-of-function methods in transcriptional analysis. Nucleic Acids Res. 46(12):5950–5966. 29860520 10.1093/nar/gky437 PMC6093183

[evaa243-B113] Sun W , Hu Y. 2013 eQTL mapping using RNA-seq data. Stat Biosci. 5(1):198–219. 23667399 10.1007/s12561-012-9068-3 PMC3650863

[evaa243-B114] Tang F , et al 2009 mRNA-Seq whole-transcriptome analysis of a single cell. Nat Methods. 6(5):377–382. 19349980 10.1038/nmeth.1315

[evaa243-B115] Trivers R. 1988 Sex differences in rates of recombination and sexual selection. Sunderland (MA): Sinauer Associates.

[evaa243-B116] Trivers RL. 1972 Sexual selection and parental investment. Chicago: Aldine Publishing Company.

[evaa243-B117] Tukiainen T , et al 2017 Landscape of X chromosome inactivation across human tissues. Nature 550(7675):244–248. 29022598 10.1038/nature24265 PMC5685192

[evaa243-B118] Turner LM , White MA, Tautz D, Payseur BA. 2014 Genomic networks of hybrid sterility. PLoS Genet. 10(2):e1004162. 24586194 10.1371/journal.pgen.1004162 PMC3930512

[evaa243-B119] Van Deursen J , Fornerod M, Van Rees B, Grosveld G. 1995 Cre-mediated site-specific translocation between nonhomologous mouse chromosomes. Proc Natl Acad Sci U S A. 92(16):7376–7380. 7638200 10.1073/pnas.92.16.7376 PMC41342

[evaa243-B120] VanInsberghe M , Zahn H, White AK, Petriv OI, Hansen CL. 2018 Highly multiplexed single-cell quantitative PCR. PLoS One 13(1):e0191601. 29377915 10.1371/journal.pone.0191601 PMC5788347

[evaa243-B121] VanKuren NW , Long M. 2018 Gene duplicates resolving sexual conflict rapidly evolved essential gametogenesis functions. Nat Ecol Evol. 2(4):705–712. 29459709 10.1038/s41559-018-0471-0 PMC5866764

[evaa243-B122] Wang H , et al 2017 cGAL, a temperature-robust GAL4-UAS system for *Caenorhabditis elegans*. Nat Methods. 14(2):145–148. 27992408 10.1038/nmeth.4109 PMC5693259

[evaa243-B123] Warnefors M , et al 2017 Sex-biased microRNA expression in mammals and birds reveals underlying regulatory mechanisms and a role in dosage compensation. Genome Res. 27(12):1961–1973. 29079676 10.1101/gr.225391.117 PMC5741053

[evaa243-B124] Wu C-C , et al 2016 Spatially resolved genome-wide transcriptional profiling identifies bmp signaling as essential regulator of zebrafish cardiomyocyte regeneration. Dev Cell. 36(1):36–49. 26748692 10.1016/j.devcel.2015.12.010

[evaa243-B125] Wyman MJ , Cutter AD, Rowe L. 2012 Gene duplication in the evolution of sexual dimorphism. Evolution 66(5):1556–1566. 22519790 10.1111/j.1558-5646.2011.01525.x

[evaa243-B126] Yamaguchi N , Colak-Champollion T, Knaut H. 2019 zGrad is a nanobody-based degron system that inactivates proteins in zebrafish. eLife 8:4640. 10.7554/eLife.43125 PMC6384026 30735119

[evaa243-B127] Yan F , Powell DR, Curtis DJ, Wong NC. 2020 From reads to insight: a hitchhiker’s guide to ATAC-seq data analysis. Genome Biol. 21(1):22. 32014034 10.1186/s13059-020-1929-3 PMC6996192

[evaa243-B128] Yang X. 2006 Tissue-specific expression and regulation of sexually dimorphic genes in mice. Genome Res. 16(8):995–1004. 16825664 10.1101/gr.5217506 PMC1524872

[evaa243-B129] Zhang L , Ward JD, Cheng Z, Dernburg AD. 2015 The auxin-inducible degradation (AID) system enables versatile conditional protein depletion in *C. elegans*. Development 142(24):4374–4384. 26552885 10.1242/dev.129635 PMC4689222

[evaa243-B130] Zhao Q , et al 2019 Genome-wide profiling of the alternative splicing provides insights into development in *Plutella xylostella*. BMC Genomics 20(1):463–414. 31174467 10.1186/s12864-019-5838-3 PMC6556048

[evaa243-B131] Zhou Q , et al 2019 Single-cell RNA-seq reveals distinct dynamic behavior of sex chromosomes during early human embryogenesis. Mol Reprod Dev. 86(7):871–882. 31094050 10.1002/mrd.23162

