# Supplementary material for: Sexual Dimorphism through the Lens of Genome Manipulation, Forward Genetics, and Spatiotemporal Sequencing
Source: Genome Biol Evol. 2020 Nov 18;13(2):evaa243. doi: 10.1093/gbe/evaa243 (PMC7883666; doi:10.1093/gbe/evaa243)
Supplement: evaa243_Supplementary_Data [file evaa243_supplementary_data.pdf]

Supplemental Table 1. Next-generation molecular techniques for identifying the basis of sexual dimorphism. For each technique, we provide a location within the genotype to phenotype (GP Map; Box 1), and brief description of the method. Additionally, we provide references for studies which utilize the technology or an applicable species-specific review for major model organisms and prominent evolutionary organisms: mammals (M: apes, humans, mouse); birds (B: chicken); fish (F: stickleback, zebrafish); amphibians (Am: frogs); arthropods (Ar: *Drosophila* sp., mosquitoes); nematodes (N: *Caenorhabditis* sp.); plants (P: *Arabidopsis* sp., corn, rice). For genome sequencing approaches, we only highlight recent reviews as these approaches are generalizable to any system in which high molecular weight DNA can be extracted. While these lists are not comprehensive, they highlight the general feasibility of these technologies across taxa.

**Technique**	**GP Map**	**Description**	**Species**
Transgenic Approaches			
Insertion	• Transcription<br>• Translation<br>• Fitness	CRISPR – targeted nuclease creates a double-strand break, which then is repaired with a synthetic construct to insert specific genes or sequences by HDR[1]	• M: *Homo sapiens*[2], *Mus musculus*[3,4], *Macaca* sp.[5], *Callithrix* sp.[5]<br>• B: *Gallus gallus*[6,7]<br>• F: *Danio rerio*[8]<br>• Am: *Xenopus* sp.[9]<br>• Ar: *D. melanogaster*[10,11], *Anopheles gambiae*[12] *1*<br>• N: *C. elegans*[13-16], *C. remanei*[17], *C. briggsae*[18]<br>• P: *A. thaliana*[19], *Oryza sativa*[20], *Zea mays*[21]
Knockdown	• Fitness<br>• Translation	CRISPRi – dCas9 binds promoter sequences blocking transcription[22]	• M: *H. sapiens*[23], *M. musculus*[4,24]<br>• B: *G. gallus*[25]<br>• F: *D. rerio*[26]<br>• Ar: *D. melanogaster*[11], *A. gambiae*[27] *1*<br>• N: *C. elegans*[26]<br>• P: *Zea mays*[28]
		RNAi – degrades native mRNA in the presence of complementary double-strand RNA[29-31]	• M: *H. sapiens*[32,33], *M. musculus*[34], *Pan troglodytes*[35]<br>• B: *G. gallus*[36]<br>• F: *D. rerio*[37]<br>• Am: *Xenopus laevis*[38]<br>• Ar: *D. melanogaster*[39], *A. gambiae*[40] *1*<br>• N: *C. elegans*[41], *C. remanei*[42], *C. briggsae*[43]<br>• P: *A. thaliana*[44], *Oryza sativa*[45], *Zea mays*[46]

Technique	GP Map	Description	Species
Transgenic Approaches continued			
Knockdown *cont.*	• Fitness<br>• Translation	Auxin – requires specific degron tag, expression of TIR1, and presence of indole-3-acetic acid (auxin)[47]	• M: *H. sapiens*[48], *M. musculus*[49]<br>• B: *G. gallus*[47]<br>• Ar: *D. melanogaster*[50] *1*<br>• N: *C. elegans*[51]
Knockout	• Processing<br>• Translation<br>• Fitness	CRISPR – targeted nuclease which can introduce indels by NHEJ or knockout specific genes by HDR[1]	• M: *H. sapiens*[52], *M. musculus*[3,4], *Macaca* sp.[5], *Callithrix* sp.[5]<br>• B: *G. gallus*[53]<br>• F: *D. rerio*[54], *Gasterosteus aculeatus*[55,56]<br>• Am: *Xenopus* sp.[9]<br>• Ar: *D. melanogaster*[11,57], *A. gambiae*[58] *1*<br>• N: *C. elegans*[13,59], *C. remanei*[17], *C. briggsae*[60]<br>• P: *A. thaliana*[61], *Oryza sativa*[62], *Zea mays*[63]
		Cre/Lox – the loxp-flanked sequence is removed upon expression of Cre-recombinase[64-66]	• M: *H. sapiens*[67], *M. musculus*[68]<br>• B: *G. gallus*[69]<br>• F: *D. rerio*[70]<br>• Am: *X. laevis*[71]<br>• Ar: *D. melanogaster*[72,73], *A. gambiae*[74] *1*<br>• N: *C. elegans*[75]<br>• P: *A. thaliana*[76], *Oryza sativa*[77], *Zea mays*[78]
Ectopic & Altered Expression	• Regulation<br>• Transcription<br>• Fitness	Gal4/UAS – UAS upstream sequence is bound by ectopic expression of Gal4, which initiates transcription{Laughon:1984gr, Ptashne:1988kf}	• M: *H. sapiens*[79], *M. musculus*[80]<br>• F: *D. rerio*[81]<br>• Am: *X. tropicalis*[82]<br>• Ar: *D. melanogaster*[83], *A. aegypti*[84] *1*<br>• N: *C. elegans*[85]<br>• P: *A. thaliana*[86], *Oryza sativa*[87], *Zea mays*[88]
		CRISPR – dCas9 is fused to a transcription activator domain and targeted to the upstream regulatory sequence of a specific genes[22]	• M: *H. sapiens*[23], *M. musculus*[89]<br>• F: *D. rerio*[26]<br>• B: *G. gallus*[25]<br>• Ar: *D. melanogaster*[90] *1*<br>• N: *C. elegans*[26]<br>• P: *A. thaliana*[91], *Zea mays*[28]

Technique	GP Map	Description	Species
Transcriptomic Approaches			
scRNA-seq	• Transcription<br>• Between cell types	Integrates single-cell isolation (cell-sorting), cDNA amplification, and highly multiplexed next-generation sequencing[92]	• M: *H. sapiens*[93-95], *M. musculus*[96,97]<br>• B: *G. gallus*[98]<br>• F: *D. rerio*[99]<br>• Am: *X. tropicalis*[100]<br>• Ar: *Drosophila* sp.[101], *A. gambiae*[102]*1*<br>• N: *C. elegans*[103]<br>• P: *A. thaliana*[104], *Oryza sativa*[105], *Zea mays*[106]
Tomo-seq	• Transcription<br>• Between tissue segments	Sequential RNA sequencing of contiguous microtome-cryo-sliced whole-body segments[107]	• M: *M. musculus*[108]<br>• F: *D. rerio*[109]<br>• Am: *X. laevis*[110]<br>• Ar: *D. melanogaster*[111]*1*<br>• N: *C. elegans*[112]
Iso-seq	• Processing	Generates full-length cDNA sequences to characterize transcript isoforms	• M: *H. sapiens*[113], *M. musculus*[114], *Macaca mulatta*[115]
ATAC-seq	• Transcription initiation<br>• Between cell types	Assay for transposase-accessible chromatin sequencing: enzymatic access to open chromatin with hyperactive Tn5 transposase. Can be coupled with single-cell applications[116]	• M: *H. sapiens*[117,118], *M. musculus*[119], *M. mulatta*[120]<br>• B: *G. gallus*[121]<br>• F: *D. rerio*[122]<br>• Am: *Xenopus* sp.[123]<br>• Ar: *D. melanogaster*[124,125]*1*<br>• N: *C. elegans*[126]<br>• P: *A. thaliana*[127], *Oryza sativa*[128]
sc-qPCR	• Transcription<br>• Between cell types	Integrates single-cell isolation (cell-sorting) and retro-transcription quantitative PCR[129]	• M: *H. sapiens*[130]
Genomic Approaches			
Long-read Sequencing	• Genotype	DNA sequencing that yields read lengths of 10 kb or higher without requiring DNA amplification[131]	• Applicable in any organisms where high molecular weight can be extracted. Reviewed in Amarasinghe et al.[131], Kono & Arakawa[132], and Logsdon et al.[133].

Technique	GP Map	Description	Species
Genomics Approaches continued			
Linked-read Sequencing	• Genotype	High molecular weight DNA molecules are attached to uniquely barcoded beads using Tn5 transposase. After sequencing barcodes are used to reconstruct maternal and paternal haplotypes of lengths over 10 kb{Chen:2020et, Wang:2019fo, Lutgen:2020ij}	• Applicable in any organisms where high molecular weight can be extracted. Reviewed in De Coster & Can Broeckhoven[134] and Ho et al.[135].

Supplemental Literature Cited

1. Doudna, J. A. & Charpentier, E. Genome editing. The new frontier of genome engineering with CRISPR-Cas9. *Science* **346,** 1258096 (2014).
2. Santos, D. P., Kiskinis, E., Eggan, K. & Merkle, F. T. Comprehensive Protocols for CRISPR/Cas9-based Gene Editing in Human Pluripotent Stem Cells. *Curr Protoc Stem Cell Biol* **38,** 5B.6.1–5B.6.60 (2016).
3. Wang, H. *et al.* One-Step Generation of Mice Carrying Mutations in Multiple Genes by CRISPR/Cas-Mediated Genome Engineering. *Cell* **153,** 910–918 (2013).
4. Singh, P., Schimenti, J. C., Genetics, E. B.-F.2015. A mouse geneticist's practical guide to CRISPR applications. *Genetics* **199,** 1–15 (2015).
5. Kang, Y., Chu, C., Wang, F. & Niu, Y. CRISPR/Cas9-mediated genome editing in nonhuman primates. *Dis. Model. Mech.* **12,** 1–7 (2019).
6. Antonova, E. *et al.* Successful CRISPR/Cas9 mediated homologous recombination in a chicken cell line. *F1000Res* **7,** 238–21 (2018).
7. Chojnacka-Puchta, L. & Sawicka, D. CRISPR/Cas9 gene editing in a chicken model: current approaches and applications. *J. Appl. Genetics* **61,** 221–229 (2020).
8. Collery, R. F. & Link, B. A. Precise Short Sequence Insertion in Zebrafish Using a CRISPR/Cas9 Approach to Generate a Constitutively Soluble Lrp2 Protein. *Front Cell Dev Biol* **7,** 167 (2019).
9. Aslan, Y., Tadjuidje, E., Zorn, A. M. & Cha, S.-W. High-efficiency non-mosaic CRISPR-mediated knock-in and indel mutation in F0 Xenopus. *Development* **144,** 2852–2858 (2017).
10. Gratz, S. J. *et al.* Highly specific and efficient CRISPR/Cas9-catalyzed homology-directed repair in *Drosophila*. *Genetics* **196,** 961–971 (2014).
11. Bier, E., Harrison, M. M., O'Connor-Giles, K. M. & Wildonger, J. Advances in Engineering the Fly Genome with the CRISPR-Cas System. *Genetics* **208,** 1–18 (2018).
12. Kyrou, K. *et al.* A CRISPR–Cas9 gene drive targeting doublesex causes complete population suppression in caged *Anopheles gambiae* mosquitoes. *Nature Biotechnology* **36,** 1062–1066 (2018).
13. Nance, J. & Frøkjær-Jensen, C. The *Caenorhabditis elegans* Transgenic Toolbox. *Genetics* **212,** 959–990 (2019).
14. Stevenson, Z. C., Moerdyk-Schauwecker, M. J., Jamison, B. & Phillips, P. C. Rapid self-selecting and clone-free integration of transgenes into engineered CRISPR safe harbor locations in *Caenorhabditis elegans*. *G3* **10,** 3775–3782 (2020).
15. Paix, A., Schmidt, H. & Seydoux, G. Cas9-assisted recombineering in *C. elegans*: genome editing using in vivo assembly of linear DNAs. *Nucleic Acids Research* **44,** e128 (2016).
16. Dokshin, G. A., Ghanta, K. S., Piscopo, K. M. & Mello, C. C. Robust genome editing with short single-stranded and long, partially single-stranded DNA donors in *Caenorhabditis elegans*. *Genetics* **210,** 781–787 (2018).
17. Yin, D. *et al.* Rapid genome shrinkage in a self-fertile nematode reveals sperm competition proteins. *Science* **359,** 55–61 (2018).
18. Cohen, S. & Sternberg, P. Genome editing of *Caenorhabditis briggsae* using CRISPR/Cas9 co-conversion marker *dpy-10*. *MicroPubl Biol* **2019,** 1–2 (2019).
19. Miki, D., Zhang, W., Zeng, W., Feng, Z. & Zhu, J.-K. CRISPR/Cas9-mediated gene targeting in *Arabidopsis* using sequential

transformation. *Nat Commun* **9,** 1–9 (2018).
20. Dong, O. X. *et al.* Marker-free carotenoid-enriched rice generated through targeted gene insertion using CRISPR-Cas9. *Nat Commun* **11,** 1–10 (2020).
21. Svitashev, S. *et al.* Targeted mutagenesis, precise gene editing, and site-specific gene insertion in maize using Cas9 and guide RNA. *Plant Physiol.* **169,** 931–945 (2015).
22. Pickar-Oliver, A. & Gersbach, C. A. The next generation of CRISPR-Cas technologies and applications. *Nat. Rev. Mol. Cell Biol.* **20,** 490–507 (2019).
23. Zhang, J., Späth, S. S. & Katz, S. G. Genome-wide CRISPRi/a screening in an *in vitro* coculture assay of human immune cells with tumor cells. *Methods Mol. Biol.* **2097,** 231–252 (2020).
24. MacLeod, R. S. *et al.* Effective CRISPR interference of an endogenous gene via a single transgene in mice. *Scientific Reports 2018 8:1* **9,** 17312–12 (2019).
25. Williams, R. M. *et al.* Genome and epigenome engineering CRISPR toolkit for *in vivo* modulation of cis-regulatory interactions and gene expression in the chicken embryo. *Development* **145,** 1–12 (2018).
26. Long, L. *et al.* Regulation of transcriptionally active genes via the catalytically inactive Cas9 in *C. elegans* and *D. rerio*. *Nat Rev Genet* **25,** 638–641 (2015).
27. Kulkarni, A. *et al.* Programmable CRISPR interference for gene silencing using Cas13a in mosquitoes. *J Genomics* **8,** 30–36 (2020).
28. Gentzel, I. N. *et al.* A CRISPR/dCas9 toolkit for functional analysis of maize genes. *Plant Methods* **16,** 133–9 (2020).
29. Fire, A. *et al.* Potent and specific genetic interference by double-stranded RNA in *Caenorhabditis elegans*. *Nature* **391,** 806–811 (1998).
30. Dzitoyeva, S., Dimitrijevic, N. & Manev, H. Intra-abdominal injection of double-stranded RNA into anesthetized adult *Drosophila* triggers RNA interference in the central nervous system. *Mol. Psychiatry* **6,** 665–670 (2001).
31. Crotty, S. & Pipkin, M. E. *In vivo* RNAi screens: concepts and applications. *Trends Immunol.* **36,** 315–322 (2015).
32. Davis, M. E. *et al.* Evidence of RNAi in humans from systemically administered siRNA via targeted nanoparticles. *Nature* **464,** 1067–1070 (2010).
33. Yang, C., Qiu, L. & Xu, Z. Specific gene silencing using RNAi in cell culture. *Methods Mol. Biol.* **793,** 457–477 (2011).
34. Premsrirut, P. K. *et al.* A rapid and scalable system for studying gene function in mice using conditional rna interference. *Cell* **145,** 145–158 (2011).
35. Wooddell, C. I. *et al.* RNAi-based treatment of chronically infected patients and chimpanzees reveals that integrated hepatitis B virus DNA is a source of HBsAg. *Sci Transl Med* **9,** eaan0241 (2017).
36. Dai, F., Yusuf, F., Farjah, G. H. & Brand-Saberi, B. RNAi-induced targeted silencing of developmental control genes during chicken embryogenesis. *Dev Biol* **285,** 80–90 (2005).
37. Andrews, O. E., Cha, D. J., Wei, C. & Patton, J. G. RNAi-mediated gene silencing in zebrafish triggered by convergent transcription. *Scientific Reports 2018 8:1* **4,** 5222 (2014).
38. Zhou, Y., Ching, Y.-P., Kok, K. H., Kung, H.-F. & Jin, D.-Y. Post-transcriptional suppression of gene expression in *Xenopus* embryos by small interfering RNA. *Nucleic Acids Res.* **30,** 1664–1669 (2002).
39. Heigwer, F., Port, F. & Boutros, M. RNA Interference (RNAi) Screening in *Drosophila*. *Genetics* **208,** 853–874 (2018).
40. Chen, J., Lu, H.-R., Zhang, L., Liao, C.-H. & Han, Q. RNA interference-mediated knockdown of 3, 4-dihydroxyphenylacetaldehyde

synthase affects larval development and adult survival in the mosquito *Aedes aegypti*. *Parasit Vectors* **12,** 1–11 (2019).
41. Conte, D., MacNeil, L. T., Walhout, A. J. M. & Mello, C. C. RNA Interference in Caenorhabditis elegans. *Curr Protoc Mol Biol* **109,** 26.3.1–26.3.30 (2015).
42. Nuez, I. & Felix, M.-A. Evolution of susceptibility to ingested double-stranded RNAs in *Caenorhabditis* nematodes. *PLoS ONE* **7,** e29811–12 (2012).
43. Verster, A. J., Ramani, A. K., McKay, S. J. & Fraser, A. G. Comparative RNAi screens in *C. elegans* and *C. briggsae* reveal the impact of developmental system drift on gene function. *PLoS Genetics* **10,** e1004077–15 (2014).
44. Dunoyer, P., Himber, C., Ruiz-Ferrer, V., Alioua, A. & Voinnet, O. Intra- and intercellular RNA interference in *Arabidopsis thaliana* requires components of the microRNA and heterochromatic silencing pathways. *Nat Genet* **39,** 848–856 (2007).
45. LI, L. *et al.* RNAi-mediated transgenic rice resistance to Rice stripe virus. *Journal of Integrative Agriculture* **15,** 2539–2549 (2016).
46. McGinnis, K. *et al.* Assessing the efficiency of RNA interference for maize functional genomics. *Plant Physiol.* **143,** 1441–1451 (2007).
47. Nishimura, K., Fukagawa, T., Takisawa, H., Kakimoto, T. & Kanemaki, M. An auxin-based degron system for the rapid depletion of proteins in nonplant cells. *Nat Meth* **6,** 917–922 (2009).
48. Natsume, T., Kiyomitsu, T., Saga, Y. & Kanemaki, M. T. Rapid protein depletion in human cells by auxin-inducible degron tagging with short homology donors. *CellReports* **15,** 210–218 (2016).
49. Miura, K. *et al.* Application of auxin-inducible degron technology to mouse oocyte activation with PLCζ. *J. Reprod. Dev.* **64,** 319–326 (2018).
50. Trost, M., Blattner, A. C. & Lehner, C. F. Regulated protein depletion by the auxin-inducible degradation system in *Drosophila melanogaster*. *Fly* **10,** 35–46 (2016).
51. Zhang, L., Ward, J. D., Cheng, Z. & Dernburg, A. The auxin-inducible degradation (AID) system enables versatile conditional protein depletion in *C. elegans*. *Development* **142,** 4374–4384 (2015).
52. Bauer, D. E., Canver, M. C. & Orkin, S. H. Generation of genomic deletions in mammalian cell lines via CRISPR/Cas9. *J Vis Exp* **83,** 1–10 (2014).
53. Oishi, I., Yoshii, K., Miyahara, D., Kagami, H. & Tagami, T. Targeted mutagenesis in chicken using CRISPR/Cas9 system. *Scientific Reports 2018 8:1* **6,** 23980–10 (2016).
54. Sorlien, E. L., Witucki, M. A. & Ogas, J. Efficient production and identification of CRISPR/Cas9-generated gene knockouts in the model system *Danio rerio*. *J Vis Exp* 1–10 (2018).
55. Hart, J. C. & Miller, C. T. Sequence-based mapping and genome editing reveal mutations in stickleback Hps5 cause oculocutaneous albinism and the casper phenotype. *G3* **7,** 3123–3131 (2017).
56. Xie, K. T. *et al.* DNA fragility in the parallel evolution of pelvic reduction in stickleback fish. *Science* **363,** 81–84 (2019).
57. Bassett, A. R., Tibbit, C., Ponting, C. P. & Liu, J.-L. Highly efficient targeted mutagenesis of *Drosophila* with the CRISPR/Cas9 system. *Cell Reports* **4,** 220–228 (2013).
58. Li, M., Akbari, O. S. & White, B. J. Highly efficient site-specific mutagenesis in malaria mosquitoes using CRISPR. *G3* **8,** 653–658 (2018).
59. Au, V. *et al.* CRISPR/Cas9 methodology for the generation of knockout deletions in *Caenorhabditis elegans*. *G3* **9,** 135–144 (2019).
60. Culp, E. *et al.* Genome editing in the nematode *Caenorhabditis briggsae* using the CRISPR/Cas9 system. *Biology Methods and*

Protocols **5,** 397–5 (2020).
61. Liu, Y., Gao, Y., Gao, Y. & Zhang, Q. Targeted deletion of floral development genes in *Arabidopsis* with CRISPR/Cas9 using the RNA endoribonuclease Csy4 processing system. *Hortic Res* **6,** 99–10 (2019).
62. Hu, X. *et al.* Using CRISPR-Cas9 to generate semi-dwarf rice lines in elite landraces. *Scientific Reports 2018 8:1* **9,** 19096–7 (2019).
63. Doll, N. M. *et al.* Single and multiple gene knockouts by CRISPR-Cas9 in maize. *Plant Cell Rep* **38,** 487–501 (2019).
64. Gu, H., Marth, J. D., Orban, P. C., Mossmann, H. & Rajewsky, K. Deletion of a DNA polymerase beta gene segment in T cells using cell type-specific gene targeting. *Science* **265,** 103–106 (1994).
65. Van Deursen, J., Fornerod, M., Van Rees, B. & Grosveld, G. Cre-mediated site-specific translocation between nonhomologous mouse chromosomes. *Proc. Natl. Acad. Sci.* **92,** 7376–7380 (1995).
66. Grégoire, D. & Kmita, M. Recombination between inverted loxP sites is cytotoxic for proliferating cells and provides a simple tool for conditional cell ablation. *Proc. Natl. Acad. Sci. U.S.A.* **105,** 14492–14496 (2008).
67. Sengupta, R. *et al.* Viral Cre-LoxP tools aid genome engineering in mammalian cells. *J Biol Eng* **11,** 45–9 (2017).
68. Kim, H., Kim, M., Im, S.-K. & Fang, S. Mouse Cre-LoxP system: general principles to determine tissue-specific roles of target genes. *Lab Anim Res* **34,** 147–13 (2018).
69. Leighton, P. A. *et al.* Generation of chickens expressing Cre recombinase. *Transgenic Res* **25,** 609–616 (2016).
70. Pan, X., Wan, H., Chia, W., Tong, Y. & Gong, Z. Demonstration of site-directed recombination in transgenic zebrafish using the Cre/loxP system. *Transgenic Res* **14,** 217–223 (2005).
71. Werdien, D., Peiler, G. & Ryffel, G. U. FLP and Cre recombinase function in *Xenopus* embryos. *Nucleic Acids Research* **29,** 1–6 (2001).
72. Oberstein, A., Pare, A., Kaplan, L. & Small, S. Site-specific transgenesis by Cre-mediated recombination in *Drosophila*. *Nat Meth* **2,** 583–585 (2005).
73. Nakazawa, N., Taniguchi, K., Okumura, T., Maeda, R. & Matsuno, K. A novel Cre/loxP system for mosaic gene expression in the Drosophila embryo. *Dev. Dyn.* **241,** 965–974 (2012).
74. Jasinskiene, N., Coates, C. J., Ashikyan, A. & James, A. A. High efficiency, site-specific excision of a marker gene by the phage P1 cre-loxP system in the yellow fever mosquito, *Aedes aegypti*. *Nucleic Acids Res.* **31,** e147 (2003).
75. Hubbard, E. J. A. FLP/FRT and Cre/lox recombination technology in *C. elegans*. *Methods* **68,** 417–424 (2014).
76. Van Ex, F., Verweire, D., Claeys, M., Depicker, A. & Angenon, G. Evaluation of seven promoters to achieve germline directed Cre-lox recombination in *Arabidopsis thaliana*. *Plant Cell Rep* **28,** 1509–1520 (2009).
77. Khattri, A., Nandy, S. & Srivastava, V. Heat-inducible Cre-lox system for marker excision in transgenic rice. *J Biosci* **36,** 37–42 (2011).
78. Zhang, W. *et al.* Cre/lox-mediated marker gene excision in transgenic maize (*Zea mays* L.) plants. *Theor Appl Genet* **107,** 1157–1168 (2003).
79. Ornitz, D. M., Skoda, R., Moreadith, R. W. & Leder, P. in *Oncogene and Transgenics Correlates of Cancer Risk Assessments* **4,** 155–172 (Springer, Boston, MA, 1992).
80. Ornitz, D. M., Moreadith, R. W. & Leder, P. Binary system for regulating transgene expression in mice: targeting *int-2* gene expression with yeast GAL4/UAS control elements. *Proc. Nat.l Acad. Sci.* **88,** 698–702 (1991).
81. Halpern, M. E. *et al.* Gal4/UAS transgenic tools and their application to zebrafish. *Zebrafish* **5,** 97–110 (2008).
82. Chae, J., Zimmerman, L. B. & Grainger, R. M. Inducible control of tissue-specific transgene expression in *Xenopus tropicalis* transgenic

lines. *Mech Dev* **117,** 235–241 (2002).
83. Ferveur, J.-F. Cuticular hydrocarbons: their evolution and roles in Drosophila pheromonal communication. *Behav. Genet.* **35,** 279–295 (2005).
84. Zhao, B. *et al.* Regulation of the gut-specific carboxypeptidase: a study using the binary Gal4/UAS system in the mosquito Aedes aegypti. *Insect Biochem Mol Biol* **54,** 1–10 (2014).
85. Wang, H. *et al.* cGAL, a temperature-robust GAL4-UAS system for *Caenorhabditis elegans*. *Nat Meth* **14,** 145–148 (2017).
86. Radoeva, T., Hove, ten, C. A., Saiga, S. & Weijers, D. Molecular Characterization of Arabidopsis GAL4/UAS Enhancer Trap Lines Identifies Novel Cell-Type-Specific Promoters. *Plant Physiol.* **171,** 1169–1181 (2016).
87. Johnson, A. A. T. *et al.* Spatial control of transgene expression in rice (*Oryza sativa* L.) using the GAL4 enhancer trapping system. *The Plant Journal* **41,** 779–789 (2005).
88. Tiwari, S. B., Wang, X. J., Hagen, G. & Guilfoyle, T. J. AUX/IAA proteins are active repressors, and their stability and activity are modulated by auxin. *Plant Cell* **13,** 2809–2822 (2001).
89. Matharu, N. *et al.* CRISPR-mediated activation of a promoter or enhancer rescues obesity caused by haploinsufficiency. *Science* **363,** 1–11 (2019).
90. Lin, S., Ewen-Campen, B., Ni, X., Housden, B. E. & Perrimon, N. In Vivo Transcriptional Activation Using CRISPR/Cas9 in *Drosophila*. *Genetics* **201,** 433–442 (2015).
91. Roca Paixão, J. F. *et al.* Improved drought stress tolerance in *Arabidopsis* by CRISPR/dCas9 fusion with a Histone AcetylTransferase. *Scientific Reports 2018 8:1* **9,** 1–9 (2019).
92. Haque, A., Engel, J., Teichmann, S. A. & Lönnberg, T. A practical guide to single-cell RNA-sequencing for biomedical research and clinical applications. *Genome Med* **9,** 75–12 (2017).
93. Kulkarni, A., Anderson, A. G., Merullo, D. P. & Konopka, G. Beyond bulk: a review of single cell transcriptomics methodologies and applications. *Current Opinion in Biotechnology* **58,** 129–136 (2019).
94. Buenrostro, J. D. *et al.* Integrated single-cell analysis maps the continuous regulatory landscape of human hematopoietic differentiation. *Cell* **173,** 1535–1537 (2018).
95. Liao, J. *et al.* Single-cell RNA sequencing of human kidney. *Sci Data* **7,** 4–9 (2020).
96. Han, X. *et al.* Mapping the mouse cell atlas by microwell-seq. *Cell* **172,** 1091–1097 (2018).
97. Skelly, D. A. *et al.* Single-cell transcriptional profiling reveals cellular diversity and intercommunication in the mouse heart. *Cell Reports* **22,** 600–610 (2018).
98. Estermann, M. A. *et al.* Insights into gonadal sex differentiation provided by single-cell transcriptomics in the chicken embryo. *Cell Reports* **31,** 1–25 (2020).
99. Farrell, J. A. *et al.* Single-cell reconstruction of developmental trajectories during zebrafish embryogenesis. *Science* **360,** eaar3131 (2018).
100. Briggs, J. A. *et al.* The dynamics of gene expression in vertebrate embryogenesis at single-cell resolution. *Science* **360,** 1–9 (2018).
101. Li, H. Single-cell RNA sequencing in *Drosophila*: technologies and applications. *WIREs Dev. Biol.* **9,** e396 (2020).
102. Severo, M. S. *et al.* Unbiased classification of mosquito blood cells by single-cell genomics and high-content imaging. *Proc. Natl. Acad. Sci. U.S.A.* **115,** E7568–E7577 (2018).

103. Cao, J. *et al.* Comprehensive single-cell transcriptional profiling of a multicellular organism. *Science* **357,** 661–667 (2017).
104. Ryu, K. H., Huang, L., Kang, H. M. & Schiefelbein, J. Single-cell rna sequencing resolves molecular relationships among individual plant cells. *Plant Physiol.* **179,** 1444–1456 (2019).
105. Wang, Y., Huan, Q., Chu, X., Li, K. & Qian, W. Single-cell transcriptome analyses recapitulate the cellular and developmental responses to abiotic stresses in rice. *bioRxiv* 1–22 (2020). doi:10.1101/2020.01.30.926329
106. Nelms, B. & Walbot, V. Defining the developmental program leading to meiosis in maize. *Science* **364,** 52–56 (2019).
107. Kruse, F., Junker, J. P., van Oudenaarden, A. & Bakkers, J. Tomo-seq: A method to obtain genome-wide expression data with spatial resolution. *Methods Cell Biol.* **135,** 299–307 (2016).
108. Lacraz, G. P. A. *et al.* Tomo-seq identifies sox9 as a key regulator of cardiac fibrosis during ischemic injury. *Circulation* **136,** 1396–1409 (2017).
109. Junker, J. P. *et al.* Genome-wide RNA Tomography in the zebrafish embryo. *Cell* **159,** 662–675 (2014).
110. Sindelka, R. *et al.* Asymmetric distribution of biomolecules of maternal origin in the Xenopus laevis egg and their impact on the developmental plan. *Scientific Reports 2018 8:1* **8,** 1–16 (2018).
111. Combs, P. A. & Eisen, M. B. Sequencing mRNA from cryo-sliced Drosophila embryos to determine genome-wide spatial patterns of gene expression. *PLoS ONE* **8,** e71820 (2013).
112. Ebbing, A. *et al.* Spatial transcriptomics of *C. elegans* males and hermaphrodites identifies sex-specific differences in gene expression patterns. *Developmental Cell* **47,** 801–813.e6 (2018).
113. Deslattes Mays, A. *et al.* Single-Molecule Real-Time (SMRT) full-length rna-sequencing reveals novel and distinct mrna isoforms in human bone marrow cell subpopulations. *Genes* **10,** 253–17 (2019).
114. Wan, Y. *et al.* Systematic identification of intergenic long-noncoding RNAs in mouse retinas using full-length isoform sequencing. *BMC Genomics* **20,** 1–15 (2019).
115. He, Y. *et al.* Long-read assembly of the Chinese rhesus macaque genome and identification of ape-specific structural variants. *Nat Commun* **10,** 4233–14 (2019).
116. Yan, F., Powell, D. R., Curtis, D. J. & Wong, N. C. From reads to insight: a hitchhiker's guide to ATAC-seq data analysis. *Genome Biol* **21,** 22 (2020).
117. Corces, M. R. *et al.* The chromatin accessibility landscape of primary human cancers. *Science* **362,** 1–15 (2018).
118. Ackermann, A. M., Wang, Z., Schug, J., Naji, A. & Kaestner, K. H. Integration of ATAC-seq and RNA-seq identifies human alpha cell and beta cell signature genes. *Molecular Metabolism* **5,** 233–244 (2016).
119. Liu, C. *et al.* An ATAC-seq atlas of chromatin accessibility in mouse tissues. *Sci Data* **6,** 65–10 (2019).
120. Yin, S. *et al.* Transcriptomic and open chromatin atlas of high-resolution anatomical regions in the rhesus macaque brain. *Nat Commun* **11,** 1–13 (2020).
121. Foissac, S. *et al.* Transcriptome and chromatin structure annotation of liver, CD4+ and CD8+ T cells from four livestock species. *bioRxiv* 1–40 (2019). doi:10.1101/316091
122. Doganli, C., Sandoval, M., Thomas, S. & Hart, D. in *Eukaryotic Transcriptional and Post-Transcriptional Gene Expression Regulation* **1507,** 59–66 (Humana Press, New York, NY, 2017).
123. Gilchrist, M. J., Cho, K. W. Y. & Veenstra, G. J. C. Genomics Methods for *Xenopus* Embryos and Tissues. *Cold Spring Harb Protoc* 1–5

(2020).
124. Blythe, S. A. & Wieschaus, E. F. Establishment and maintenance of heritable chromatin structure during early Drosophila embryogenesis. *eLife* **5,** e1003428 (2016).
125. Chen, X., Rahman, R., Guo, F. & Rosbash, M. Genome-wide identification of neuronal activity-regulated genes in *Drosophila*. *eLife* **5,** 1–21 (2016).
126. Daugherty, A. C. *et al.* Chromatin accessibility dynamics reveal novel functional enhancers in *C. elegans*. *Genome Res.* **27,** 2096–2107 (2017).
127. Lu, Z., Hofmeister, B. T., Vollmers, C., DuBois, R. M. & Schmitz, R. J. Combining ATAC-seq with nuclei sorting for discovery of cis-regulatory regions in plant genomes. *Nucleic Acids Res.* **45,** 1–13 (2016).
128. Wilkins, O. *et al.* EGRINs (Environmental Gene Regulatory Influence Networks) in rice that function in the response to water deficit, high temperature, and agricultural environments. *Plant Cell* **28,** 2365–2384 (2016).
129. VanInsberghe, M., Zahn, H., White, A. K., Petriv, O. I. & Hansen, C. L. Highly multiplexed single-cell quantitative PCR. *PLoS ONE* **13,** e0191601 (2018).
130. Hajji, I. *et al.* Droplet microfluidic platform for fast and continuous-flow RT-qPCR analysis devoted to cancer diagnosis application. *Sensors & Actuators: B. Chemical* **303,** 1–8 (2020).
131. Amarasinghe, S. L. *et al.* Opportunities and challenges in long-read sequencing data analysis. *Genome Biol* **21,** 30–16 (2020).
132. Kono, N. & Arakawa, K. Nanopore sequencing: review of potential applications in functional genomics. *Develop. Growth Differ.* **61,** 316–326 (2019).
133. Logsdon, G. A., Vollger, M. R. & Eichler, E. E. Long-read human genome sequencing and its applications. *Nat Rev Genet* **34,** 666–18 (2020).
134. De Coster, W. & Van Broeckhoven, C. Newest methods for detecting structural variations. *Trends in Biotechnology* **37,** 973–982 (2019).
135. Ho, S. S., Urban, A. E. & Mills, R. E. Structural variation in the sequencing era. *Nat Rev Genet* **21,** 171–189 (2020).
